# HDC/histamine Signaling Axis Drives Macrophage Reprogramming to Promote Angiogenesis in Hindlimb-Ischemic Mice

**DOI:** 10.7150/ijbs.105148

**Published:** 2025-03-19

**Authors:** Dili Sun, Jianfu Zhu, Gaofeng Zeng, Xiyang Yang, Xiaowei Zhu, Diyaerjiang Aierken, Zhaocheng Shi, Suling Ding, Junbo Ge, Hai Hu, Xiangdong Yang

**Affiliations:** 1 Shanghai Institute of Cardiovascular Diseases, Zhongshan Hospital, Fudan University, Shanghai, 200032, China.; 2 Department of Cardiology, Zhongshan Hospital, Fudan University, Shanghai, 200032, China.; 3 Department of Cardiology, the Second Affiliated Hospital, Hengyang Medical School, University of South China, Key Laboratory of Heart Failure Prevention & Treatment of Hengyang, Clinical Medicine Research Center of Arteriosclerotic Disease of Hunan Province, 421001, China.; 4 Department of Cardiology, Zhongshan Hospital Wusong Branch, Fudan University Shanghai, 200032, China.; 5 Department of Oral Mucosa and Periodontal Clinic, Shanghai Stomatological Hospital& School of Stomatology&Shanghai Key Laboratory of Craniomaxillofacial Development and Diseases, Fudan University, Shanghai, 200433, China.; 6 Department of Orthopedic Surgery and Orthopedic Biomechanical Laboratory, Shanghai Sixth People's Hospital Affiliated to Shanghai Jiao Tong University School of Medicine, Shanghai, 200233, China.

**Keywords:** histidine decarboxylase, histamine, macrophage, reprogramming, endothelial cells, angiogenesis

## Abstract

Histamine is catalyzed by histidine decarboxylase (HDC), which plays important roles in many physiological and pathological processes, but its role in angiogenesis has not been thoroughly clarified. Here we report that HDC is highly expressed in Ly6C^+^ macrophages, rather than in endothelial cells using Hdc-GFP transgenic mice with hindlimb ischemia (HLI) mouse model. Given the whole-process promoting effect of macrophages on angiogenesis, a cluster of HDC^+^CXCR2^+^ macrophages have been identified by single-cell sequencing technology in ischemic tissue. The inactivation of HDC leads to a lack of histamine and pro-angiogenic factor production in macrophages, inducing a harsh inflammatory microenvironment that is not conducive to the interaction between macrophages and endothelial cells. Moreover, HA-DA@histamine hydrogel has been designed and demonstrated to safely treat ischemic injury by modulating inflammation and angiogenesis. These data highlight the critical roles of HDC/histamine signaling in macrophage differentiation, angiogenesis, and muscle regeneration in the early stage of HLI.

## Introduction

Panvascular disease is a series of vascular diseases caused by atherosclerosis, mainly involving the heart, brain, kidney arteries and lower limb arteries 1, 2. Panvascular disease is characterized by high incidence, high disability rate and high case fatality rate. Delayed revascularization may lead to ischemic damage of vital organs and elevate the likelihood of experiencing adverse cardiovascular events (MACE), such as myocardial infarction, stroke, amputation, and cardiovascular death 3. Increased angiogenesis and subsequent enhancement of blood flow are crucial factors in the recovery process following ischemic injury 4, 5, although clinical utilization of treatments aimed at this mechanism remains infrequent yet. A comprehensive understanding of the underlying mechanisms of angiogenesis and ischemic revascularization is essential in order to enhance the efficacy of future clinical interventions 6, 7.

The process of angiogenesis is characterized by a sequence of intricate events, such as the degradation of the basement membrane, the migration and proliferation of endothelial cells (ECs), the formation of tubes, the fusion of newly formed vessels, and the stabilization by support cells like pericytes, which significantly influenced by the interplay of pro- and anti-angiogenic signals 8, 9. Thus, angiogenesis is a complex and coordinated process that depends on the interplay and control of various cells and molecules within the vascular microenvironment. Macrophages, known for their exceptional diversity and phenotypic plasticity 10, participate in the whole process of angiogenesis with the capacity to production of cytokines, growth factors, and proteolytic enzymes 11. For example, M1 phenotype macrophages stimulated by lipopolysaccharide (LPS) and interferon-γ (IFN-γ) secrete vascular endothelial growth factor A (VEGFA), a potent inducer of angiogenesis 12. M2a macrophages, when activated by Th2 cytokines interleukin-4 (IL-4) and IL-13, play a crucial role in the stabilization of newly formed blood vessels by secreting platelet-derived growth factor-BB (PDGF-BB) 13. M2c macrophages are activated by IL-10 and produce elevated levels of matrix metalloproteinases (MMPs) 14, while M2f macrophages are activated through the phagocytosis of apoptotic cells (efferocytosis) and release anti-inflammatory mediators 15. Different subtypes of macrophages create a microenvironment for angiogenesis through precise coordination and secretion of angiogenic proteins, thereby promoting the formation of vascular ecological niches.

Histamine is catalyzed from L-histidine by histidine decarboxylase (HDC), which plays important roles in allergy, gastric acid secretion, cell differentiation, inflammatory response, and tumorigenesis 15, 16. Previous research indicated that HDC exhibits elevated levels of expression in CD11b^+^Gr-1^+^ immature myeloid cells located in the bone marrow and these HDC-expressing cells could be mobilized to inflammatory sites and differentiated into Ly6G^+^ neutrophils in colonic carcinogenesis 17. A recent study revealed that histamine deficiency in Hdc knockout mice (Hdc^-/-^) represses myoblast proliferation and retards ischemic skeletal muscle regeneration via a H_3_R dependent signaling pathway 18. Histamine has both vasoconstrictive and vasodilatory effects 17, 19. However, there is limited understanding of the function of HDC^+^ macrophages and histamine signal in the context of limb ischemia and vessel revascularization.

In this research, we used single-cell RNA sequencing (scRNA-seq) data to analyze deeply the characteristics and interactions of various macrophage subsets present in the ischemic microenvironment and identified a novel macrophage subset with high levels expression of HDC, CXCR2, and angiogenic genes in the hindlimb ischemia (HLI) mouse model. Therefore, we used Hdc-GFP transgenic mice to create HLI model and demonstrated a large number of the bone marrow-derived HDC^+^ macrophages infiltrated in the injured limb. Furthermore, histamine deficient Hdc^-/-^ mice were used to establish HLI model, bone marrow transplantation (BMT) model, and macrophage differentiation model to explore the underlying mechanisms. We explored the crucial functions of HDC^+^ macrophages and histamine they secreted in regulating the interactions between endothelial cells and HDC^+^ macrophages during angiogenesis of HLI mice. This study offers a unique perspective on the precise mechanism through which HDC^+^ macrophages contribute to the process of functional restoration following ischemic events. In addition, to address the issue of stable supply of histamine within the inflammatory microenvironment, an injectable Dopamine (DA)-crosslinked hyaluronan (HA) hydrogel has been designed to efficiently deliver histamine locally in ischemic tissues. Thus, this study not only discovered and elucidated the mechanism by which HDC^+^ macrophages promote angiogenesis after HLI, but also formed a new transformable strategy for revascularization of lower extremity after ischemic injury.

## Materials and Methods

### Mice

Professor Timothy C. Wang of Columbia University graciously supplied Hdc-GFP and Hdc knockout (Hdc^-/-^) mice for the purposes of this research. The Hdc-GFP and Hdc^-/-^ mice utilized in this study are of the Balb/C genetic background. Regarding the use of Hdc-GFP transgenic mice and Hdc^-/-^ mice, previous studies have demonstrated that HDC is not expressed in vascular endothelial cells, muscle cells, or cardiomyocytes. Instead, HDC is highly expressed in CD11b^+^Ly6C^+^ monocytes/macrophages and CD11b^+^Ly6G^+^ neutrophils within the bone marrow and these myeloid cells could be released into the circulation in response to stimuli 17,18. Using Hdc^-/-^ mice combined with bone marrow transplantation (BMT) experiments, along with flow cytometry or classical bone marrow-derived macrophage culture methods, high-quality CD11b^+^Ly6C^+^ monocytes/macrophages could be efficiently obtained from Hdc^-/-^ mice, and their phenotype is similar to macrophage-specific HDC conditional knockout mice in some experiments. To establish appropriate experimental controls, Balb/C mice were obtained from the Department of Laboratory Animal Science at Fudan University Table S1. A specific pathogen-free environment was provided at Fudan University's Animal Care Facility for the mice. The study involving animals adhered to the guidelines for the care and utilization of laboratory animals and received approval from the Committee on the Ethics of Animal Experiments at Fudan University (approval reference number: SYXK 2021-0022).

### HLI model

The process of creating animal models was detailed in a prior publication 20. The animals were administered 2.5% isoflurane via intubation for anesthesia before being positioned supine on the preoperative table. In order to effectively eliminate hair from the hindlimb, a depilatory cream was administered. The femoral artery was successfully located, leading to the exposure of the neurovascular bundle. The arterial and venous vessels were dissected from the nerve. A 6-0 silk suture was used to tie off the right femoral artery above the inguinal ligament and below the popliteal artery, after which the iliofemoral artery and vein segments between the ligatures were excised. The animal was positioned on a heated pad and subjected to continuous monitoring until regaining consciousness following the closure of the incision using an absorbable suture. In order to confirm the presence of ischemia in the limb, laser Doppler blood perfusion analysis was conducted.

### Laser Doppler perfusion imaging

Utilizing a Laser Doppler perfusion imager (MoorLDI2-HIR), noninvasive assessments of hindlimb perfusion were conducted at various time points, including pre-ligation and at 1, 7, 14, and 21 days post-ligation. A color-coded image representing relative perfusion levels was visually displayed on a video monitor during sequential scanning of the hindlimbs. Perfusion was displayed as blue for poor or no perfusion, and red for high perfusion. mLDIMainV53 software was used to evaluate all images. To address the potential influence of light and temperature, the perfusion rates of limbs were compared between the ischemic limb (IL) and non-ischemic limb (NIL) on the right and left sides, respectively, as a means of mitigation.

### Assessment of limb ischemic damage

To assess ischemic damage, limb functional scores (0 indicates the same as the control ateral hindlimb; 1 indicates mild discoloration; 2 indicates moderate discoloration; 3 indicates severe discoloration; 4 indicates an amputation); and ambulatory impairment scores (0 indicates toe flexion; 1 indicates plantar flexion; no dragging of foot; 2 indicates dragging; 3 indicates dragging) were used. Higher scores indicated more severe impairments in limb function.

### BM transplantation

To prepare the suspension for BM transplantation (BMT), bone marrow cells were extracted from the femurs and tibias of donor mice, isolated into individual cells using a 40-micron cell filter, and subsequently reconstituted in sterile phosphate-buffered saline (PBS) (Sangon Biotech). After receiving 9.0 gray of radiation, recipients were injected with a suspension containing 5 x 10^6^ donor BM cells though the tail vein. Four weeks after bone marrow transplantation, mice bone marrow cells are rebuilt and could be used to establish the lower limb ischemia model in this study.

### Cell isolation and culture

Bone marrow-derived macrophages (BMDMs) were obtained by isolating bone marrow from healthy 8-12-week Balb/C mice, Hdc-GFP, and Hdc^-/-^ mice, followed by the preparation of a single cell suspension through femur washing with PBS. Following the dissolution of red blood cells using lysis buffer (BD Biosciences), the cells were subjected to *in vitro* stimulation with 20 ng/ml M-CSF (PeproTech) for a period of 5-7 days to promote the differentiation of cells into BMDMs. BMDMs were stimulated to differentiate into M1 macrophages by exposure to 100 ng/mL LPS (Sigma) and 20 ng/mL IFN-γ (PreproTech) or into M2 macrophages by treatment with 25 ng/mL IL-4 (PreproTech) and 25 ng/mL IL-13 (PreproTech).

Human umbilical cord vein endothelial cells (HUVECs) were obtained by treating the umbilical vein with a collagenase solution at 37℃ for a duration of 15 minutes. HUVECs were cultured in endothelial cell medium (ECM) under controlled conditions of a humidified incubator with 5% carbon dioxide. H_2_O_2_ was added to a final concentration of 100 μmol/L to create an ischemia model.

To perform transwell co-culture, BMDMs of Balb/C and Hdc^-/-^ mice and HUVECs were inoculated on and off Corning transwell inserts (Sigma-Aldrich) with a 0.4 mm aperture, respectively. The follow-up experiment was carried out after 24 hours of culture.

### Flow cytometric analysis

Blood, spleen, bone marrow, and muscle were taken from Hdc^-/-^ and WT mice at 3 and 7 days after hind limb ischemia, respectively. The peripheral blood samples were treated with erythrocyte lysis, the spleen samples were ground, and the bone marrow samples were rinsed with PBS. Skeletal muscle samples were incubated with type I collagenase (3mg/ml) (Worthington) and neutral protease (4mg/ml) (Worthington) at 37℃ for 15 min, and then filtered with a 40μm filter. To label specific cells, conjugated antibodies (1M per 10^6^ cells) were allowed to incubate at 4°C for a duration of 60 minutes. Two groups of antibodies listed in the Supplemental information were used to examine immune cells and endothelial cells Table S2.

### Immunofluorescence and immunohistochemistry assay

For immunofluorescence assay, the mouse gastrocnemius tissue was preserved by fixation in 4% paraformaldehyde and subsequent embedding in optimal cutting temperature compound (OCT). After freezing, it is cut into seven-meter slices at -25°C. A 12-well plate with glass coverslips was used for the culture of BMDMs and HUVECs. 4% paraformaldehyde was used as a fixative and 0.2% Triton-X was used as a permeant. The primary antibody was allowed to incubate overnight at 4°C, followed by incubation with a secondary antibody at 37°C for 1 hour following PBS washing. The nuclei were labeled with DAPI. For immunohistochemistry assay, mouse gastrocnemius tissue was fixed with 4% formalin, paraffin embedded and sliced for H&E, Masson, and immunohistochemistry staining. The antibodies are detailed in the Supplementary Materials Table S3.

### Western blot analysis

A mixture of proteinase and phosphatase inhibitor cocktails was added to ice cold lysis buffer before homogenization of muscles and cells. The BCA Protein Assay Kit (Beyotime) was utilized to determine protein concentrations, followed by the separation of equal quantities of total protein using 10% SDS-polyacrylamide gel electrophoresis and subsequent transfer onto polyvinylidene difluoride membranes. The primary antibody was incubated overnight at 4 °C, followed by the application of a secondary antibody conjugated with horseradish peroxidase (HRP) at 37°C. The blots were then visualized using an ChemiScope chemiluminescence imaging system. The Supplemental Information comprises a detailed inventory of the principal antibodies employed in the present investigation Table S3.

### RNA extraction and quantitative RT-PCR

RNA was extracted from gastrocnemius muscles, BMDMs, and HUVECs using TRIzol reagent (Takara). Reverse transcription was conducted utilizing the Prime ScriptTM RT reagent Kit (Takara), followed by amplification using the TB Green TM Premix Ex Taq TM Kit (Takara) with the CFX Connect TM Real-Time System (Bio-Rad). Using GAPDH control values as a standard, final results were calculated from independent values for each gene. The primer sequences included in the Supplemental Information were designed using sequences obtained from the GeneBank databases available on PubMed and were synthesized by Huagene Biotech Table S4.

### ELISA

Histamine levels in serum and tissue homogenates were measured using an ELISA kit (DLD Diagnostika) and VarioskanLux (thermos scientific). Additionally, the concentrations of IL-1β, IL-6, VEGF, and MMP-9 were determined using an enzyme-linked immunosorbent assay kit (BOSTER) and VarioskanLux (thermos scientific).

### Batch-corrected integrated scRNA-seq analysis

The primary analysis of the obtained single-cell RNA-sequencing (scRNA-seq) data was performed utilizing the R package Seurat (version 4.4.0) 21. Cell filtering criteria were applied, whereby only cells with a minimum detected gene count of 200 and genes detected in at least three cells were retained for downstream analysis. Subsequently, cells exhibiting over 5% mitochondrial genes or 50% ribosomal genes were excluded. The gene counts were scrutinized utilizing the "isOutlier" function from the R package Scater (version 1.30.1). Additionally, potential doublets were identified and filtered using the R package scDblFinder (version 1.14.0) with a threshold set at 0.075%. Following quality control measures, a grand total of 42,751 cells were selected for further analysis.

Integration of all cells into a unified object was performed using the R package harmony (version 1.2.0). Subsequently, the R package decontX (version 1.0.0) was subsequently utilized to address contamination effects. Cell cycle analysis was conducted, and cell cycle-associated differences were regressed using the "CellCycleScoring" function in Seurat. Clustering of cells into distinct types was achieved utilizing the "FindClusters" function of Seurat, followed by identification of marker genes employing the "FindAllMarkers" function. For cluster annotation, the R package SingleR (version 2.2.0) was utilized, with manual verification to ensure annotation accuracy. Trajectory analysis was conducted utilizing the R package monocle3 (version 1.3.4), with HDC^+^ macrophages designated as the root based on their prevalence in the early stages of injury and CytoTRACE (version 0.3.3) 22 analysis. Furthermore, gene expression variation aligned with pseudotime and Weighted Correlation Network Analysis (WCGNA) analysis were performed using the "Track_genes" and "find_gene_modules" functions of Monocle3. Data analysis and visualization were facilitated utilizing the R package SCP (version 0.5.6).

### Receptor-ligand communication between cell types

To predict ligands produced by different subtypes of macrophages and their communication with endothelial cell receptors, the R package Nichenetr (version 2.0.6) 23 was utilized. Initially, a reference receptor-ligand matrix was constructed based on data provided by the package authors. Differentially expressed genes (DEGs) between pre-injury and post-injury endothelial cells were identified using the "FindMarkers" function of Seurat, followed by screening for matched receptors. Potential ligands were predicted using the "predict_ligand_activities" function in Nichenetr. Specific genes of interest, including Vegfa, Mmp9, Il1b, Il1a, Il15, Il16, and the top 15 genes after ligand indication, were specified as targets. Ligands associated with different macrophage subtypes were determined based on average expression calculated using the "Average Expression" function. Prediction results were visualized using the "chord Diagram" function.

### RNA-seq and data processing

The total RNA of muscles 3 days after surgery from WT and Hdc^-/-^ mice was extracted using TRIzol reagent (Takara) for RNA-seq analysis. The quality of raw sequencing reads post initial quality control (version 0.20.1) was evaluated using FastQC (version 0.11.9), followed by quantification of gene expression levels through alignment to the reference genome using HISAT2 (version 2.1.0) and analysis with HTSeq-count (version 0.11.2). Clean reads were mapped to the GRCm39 mouse reference genome using STAR (version 2.7.11b) to generate the expression count matrix. The counts matrix was converted into FPKM values to normalize the RNA-Seq data for gene length and sequencing depth. The FPKM matrix was imported into R and analyzed using the DESeq2 package (version 1.22.2) 24 to normalize the data and estimate dispersion. Determinants of DEG were those with a p-value < 0.05 and a log2 fold change > 1. Ultimately, the analysis of KEGG pathway enrichment was conducted using the cluster Profiler package (version 4.13.0) 25 in R. Visualization was done using the R packages ggplot2 (version 3.4.4) and pheatmap (version 1.0.12).

### Data sources and Statistical analysis for Mendelian randomization

Mendelian randomization (MR) was employed to examine the potential causal association between antihistamine medication and the subsequent outcomes of lower limb injuries. Genetic data were obtained from genome-wide association studies (GWAS) conducted by Finngen^26^ (finngen_R7_ST19_SEQUEL_INJURI_LOWER_LIMB) and Sakaue S *et al.* 27 (GCST90019001-EFO_0009943). SNPs were included if they had genome-wide significance (p < 5 × 10^7^), and then clumped using the "ld_clump" function in the R package ieugwasr (version 1.0.0). Next, analyses were conducted with the R package TwoSampleMR (version 0.6.2) 28. Briefly, data were converted to the same format and then harmonized. The "mr" function was utilized to produce outcomes from various MR methodologies, such as MR Egger, Weighted Median, Inverse Variance Weighted, Simple Mode, and Weighted Mode. Heterogeneity tests, horizontal pleiotropy tests, and Leave-One-Out Cross-Validation (LOO-CV) were then performed to ensure the validity of the results.

### Hydrogel preparation and characterization

HA-DA hydrogel was synthesized by a previously reported procedure 29. Briefly, the HA-DA conjugate was prepared by completely dissolubility of 1.0g hyaluronic acid (HA) in 100 mL deionized water and stored in a nitrogen environment. HA solution was slowly added with 1.48 g EDC-HCl and 0.9 g NHS and stirred vigorously for 20 minutes, followed by the addition of 1480 mg dopamine (DA) and 1000 mg histamine diphosphate, swirling vigorously for one minute. The pH of the solution was subsequently modified to a range of 5.0-5.5, and dialysis bags of 8000-14000 Da were used for 48h to completely remove unreacted reagents and salts. Hydrogel samples were examined using scanning electron microscopy (SEM) to study their morphology. In order to test the expansion rate, the hydrogel was placed in a sealed small bottle filled with PBS. Following incubation at 37℃ for a specified duration, the hydrogel was removed, with the surface water sucked away, and weighed to calculate the expansion rate. Degradation rate was measured by weighing the initial weight W0 and weight Wt of hydrogels soaked in 37℃ PBS respectively, and calculating according to the formula: degradation rate (%) = (W0-Wt) / W0 ×100%. Samples should be freeze-dried at -20 ◦C before weighing.

### Statistical analysis

Each experiment was conducted a minimum of six times, and the findings are presented as mean ± standard deviation. GraphPad Prism 9 was used for statistical analysis. Statistical comparisons between two groups were conducted using Student's t-tests, whereas comparisons involving more than two groups were performed using one-way ANOVA test. P <0.05 was considered significant for differences between two group.

## Results

### The deletion of Hdc impairs hindlimb ischemia (HLI)-induced revascularization

**Figure 1:**
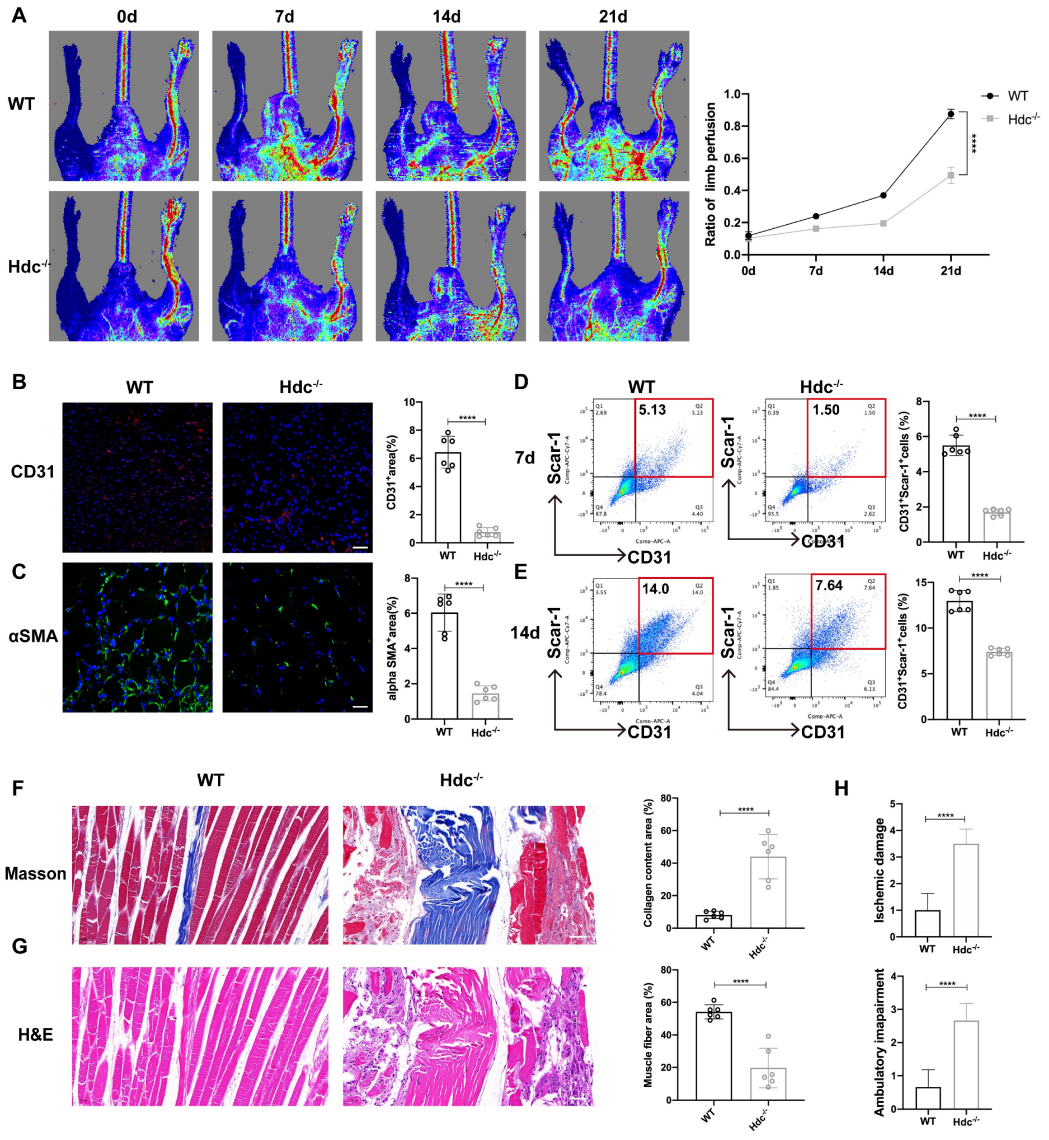
** The Deletion of Hdc Impairs Hindlimb Ischemia (HLI)-Induced Revascularization. (A)** Representative images and quantification of hindlimb blood perfusion in WT and Hdc^-/-^ mice using laser Doppler imaging at 0, 7, 14 and 21 days after femoral ligation (n=6). Red, green, and blue indicated the fastest, intermediate, and slowest blood flow velocities, respectively. **(B)** Representative images of CD31 (red), and DAPI (blue) immunostainings and quantification of CD31^+^ ECs in muscles at 3 days (n=6); scale bar, 50µm. **(C)** Representative images of αSMA (green), and DAPI (blue) immunostainings and quantification of αSMA^+^ cells in muscles at 3 days (n=6); scale bar, 50µm. **(D)** Representative flow cytometry plots with quantification of CD31^+^ Sca-1^+^ endothelial cells 7 days after HLI (n=6). **(E)** Representative flow cytometry plots with quantification of CD31^+^ Sca-1^+^ endothelial cells 14 days after HLI (n=6). **(F)** Representative images and quantitative analysis of Masson's staining of injured gastrocnemius muscle from Hdc^-/-^ and WT mice at day 21 post-injury in ischemic muscles (n=6); Scale bar, 50µm. **(G)** Representative images and quantitative analysis of H&E staining of injured gastrocnemius muscle from Hdc^-/-^ and WT mice at day 21 post-injury in ischemic muscles (n=6); Scale bar, 50µm. **(H)** Ischemic damage scores, ambulatory impairment scores in WT and Hdc^-/-^ mice (n=6). For all experiments, error bars represent the mean ± SD.*P < 0.05, **P < 0.01, ***P < 0.001, ****P < 0.0001.

The effect of HDC/histamine signaling on angiogenesis varies under different pathological conditions, and its mechanism needs to be further explored. Histamine-deficient mice (Hdc knockout, Hdc^-/-^) and wild-type mice (WT, Balb/c) were applied to establish a Hindlimb ischemic model (HLI) by the femoral artery ligation, resulting in profound ischemia-induced muscle injury. Laser Doppler imaging revealed that blood flow upon HLI was similarly reduced in both WT and Hdc^-/-^ mice at baseline, indicating that HLI models have been successfully established and histamine deficiency does not significantly alter blood flow under physiological conditions. The findings of the perfusion analysis demonstrated a notable delay in the recovery of blood flow in the ischemic limbs of Hdc^-/-^ mice in comparison to WT mice over the 21-day follow-up period (Figure 1A). Hdc^-/-^ mice exhibited a higher incidence of necrotic toes following hindlimb ischemia Figure S1A). Immunofluorescence staining with anti-CD31 demonstrated that the number of CD31^+^ endothelial cells in the ischemic muscle of Hdc^-/-^ mice significantly decreased compared to WT mice 3 days after HLI, indicating a diminished vascular density in the affected limbs of Hdc^-/-^ mice (Figure 1B). Additionally, we observed a significant presence of arterial endothelial cells that had spontaneously formed vascular networks in the injured limbs of WT mice, which were further stabilized by α-SMA^+^ (α-smooth muscle actin-positive) cells, in comparison to Hdc^-/-^ mice on day 3 (Figure 1C). Furthermore, flow cytometric analysis (FACS) data confirmed less CD31^+^Sca-1^+^ arteriole cells in the injured limbs of Hdc^-/-^ mice on day 7 and day 14 after ischemic injury compared to WT mice (Figure 1D, E).

Histological analysis was conducted to evaluate the impact of angiogenesis on the healing process of ischemic limb injury by assessing muscle damage. The images of Masson staining and hematoxylin and eosin (H&E) staining demonstrated that Hdc^-/-^ mice developed serious fibrosis and muscle necrosis compared with WT mice 21 days after HLI (Figure 1F, G). Furthermore, the limb function scores for ischemia damage and ambulatory impairment were evaluated, revealing that Hdc^-/-^ mice exhibited elevated scores in both categories, suggesting a restricted capacity for functional regeneration in ischemic limbs by day 21(Figure 1H). Collectively, these findings indicate that HDC and histamine are pivotal in the process of revascularization and skeletal muscle regeneration following HLI.

### Bone marrow-derived macrophages (BMDMs) are the predominant HDC-expressing sites during HLI

**Figure 2:**
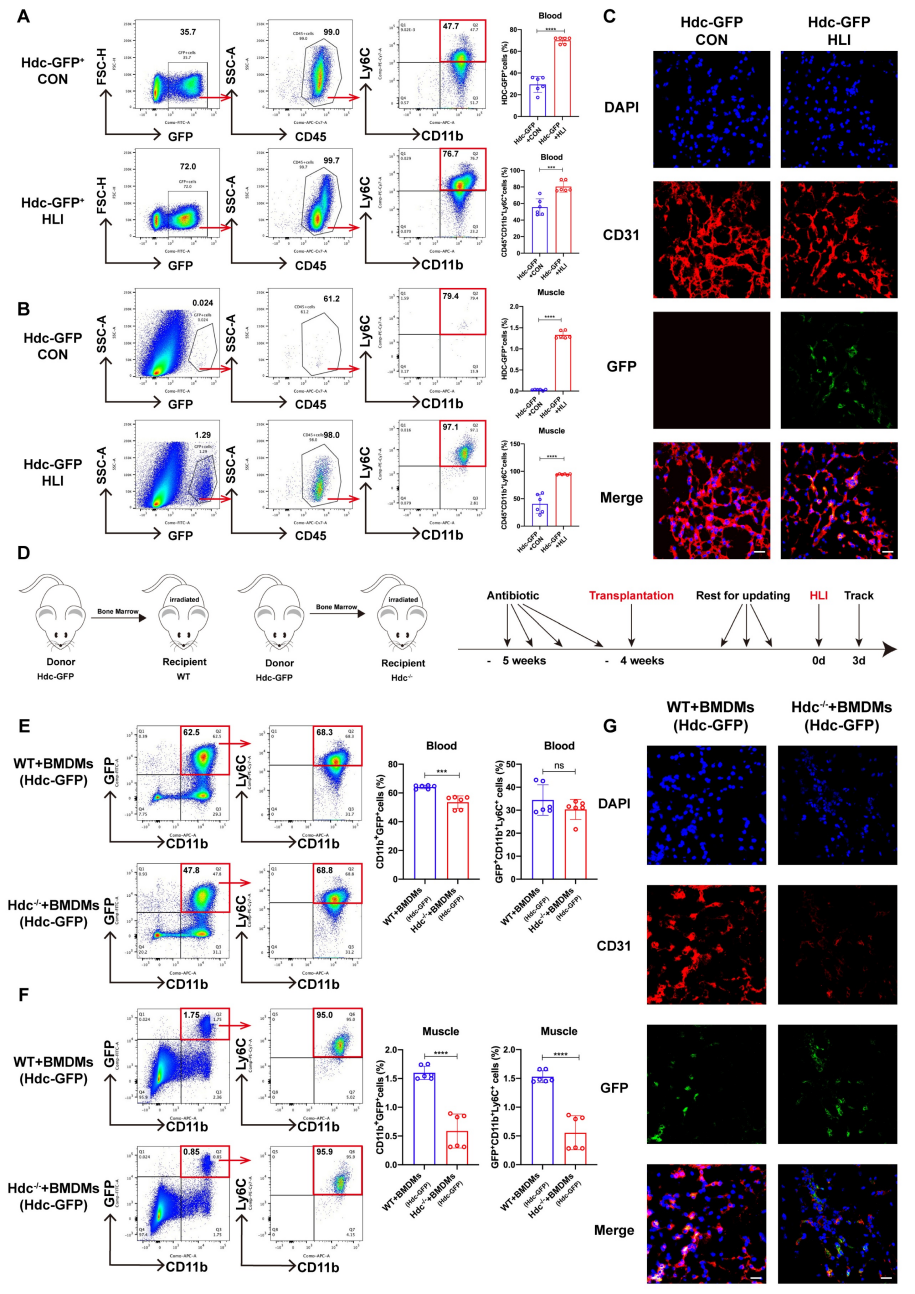
** Bone marrow-derived macrophages (BMDMs) are the predominant HDC-expressing sites during HLI. (A)** Representative images and quantification of FACS analysis of the GFP^+^ cell percentage in the peripheral blood of Hdc-GFP mice before and 3 days after HLI (n=6). **(B)** Representative images and quantification of FACS analysis of the GFP^+^ cell percentage in the muscle tissue of Hdc-GFP mice before and 3 days after HLI (n=6). **(C)** Representative images of CD31 (red), Hdc-GFP (green), and DAPI (blue) immunostainings of gastrocnemius muscle of Hdc-GFP mice before and 3 days after HLI; Scale bar, 50µm. **(D)** Scheme showing bone marrow transplantation experiment. **(E)** Representative image and quantification of FACS analysis of the GFP^+^ cell percentage in the peripheral blood of WT and Hdc^-/-^ mice transplanted with Hdc-GFP bone marrow 3 days after HLI (n=6). **(F)** Representative images and quantification of FACS analysis of the GFP^+^ cell percentage in the muscle tissue of WT and Hdc^-/-^ mice transplanted with Hdc-GFP bone marrow 3 days after HLI (n=6). **(G)** Representative images of CD31 (red), Hdc-GFP (green), and DAPI (blue) immunostainings of gastrocnemius muscle of WT and Hdc^-/-^ mice transplanted with Hdc-GFP bone marrow 3 days after HLI; Scale bar, 50µm. For all experiments, error bars represent the mean ± SD. *P < 0.05, **P < 0.01, ***P < 0.001, ****P < 0.0001.

To ascertain the cellular origin of HDC-expressing cells in mice with HLI, Hdc-GFP transgenic mice (Hdc-GFP) were utilized to establish HLI model. The findings from FACS and immunofluorescence staining revealed that GFP expression is present in CD11b^+^ myeloid cells and CD11b^+^Ly6C^+^ macrophages, rather than in skeletal muscle and vascular endothelial cells of Hdc-GFP mice with HLI (Figure 2A, B, C). RT-qPCR and Elisa data also confirmed higher levels of Hdc mRNA expression and histamine secretion in BMDMs than in endothelial cells (HUVECs) (Figure S2A, B). Furthermore, the results of FACS and immunofluorescence staining showed a significant decrease of CD31^+^endothelial cells in the ischemic muscle tissue after HLI, accompanied by a large amount of GFP^+^CD11b^+^Ly6C^+^ cell infiltration (Figure 2B, C). To further clarify the origin of these Hdc-expressing cells, FACS data verified alterations in Hdc-GFP cell populations within the bone marrow and spleen of Hdc-GFP mice, suggesting that GFP^+^ monocytes could be mobilized from the bone marrow after HLI (Figure S2C, D). In addition, the result of co-focal examination with anti-HDC demonstrated the expression of HDC located in the cytoplasm of macrophages isolated from the bone marrow of Hdc-GFP mice (Figure S2E). We finally performed bone marrow transplantation experiments (Figure 2D) and the results confirmed that GFP^+^ bone marrow-derived monocytes could be mobilized and recruited to the ischemic limbs of irradiated Hdc^-/-^ mice (Figure 2E, F). Immunofluorescence results confirmed that Hdc-GFP cells from the bone marrow of donor mice could chemotaxis to the injured muscle after HLI (Figure 2G). Thus, these results identify the bone marrow-derived macrophage subset with high level HDC expression and its dysfunction aggregates the injury of the ischemic limbs.

### Single-cell transcriptomics analysis suggests an association between HDC^+^ macrophages and angiogenesis during the process of HLI

**Figure 3:**
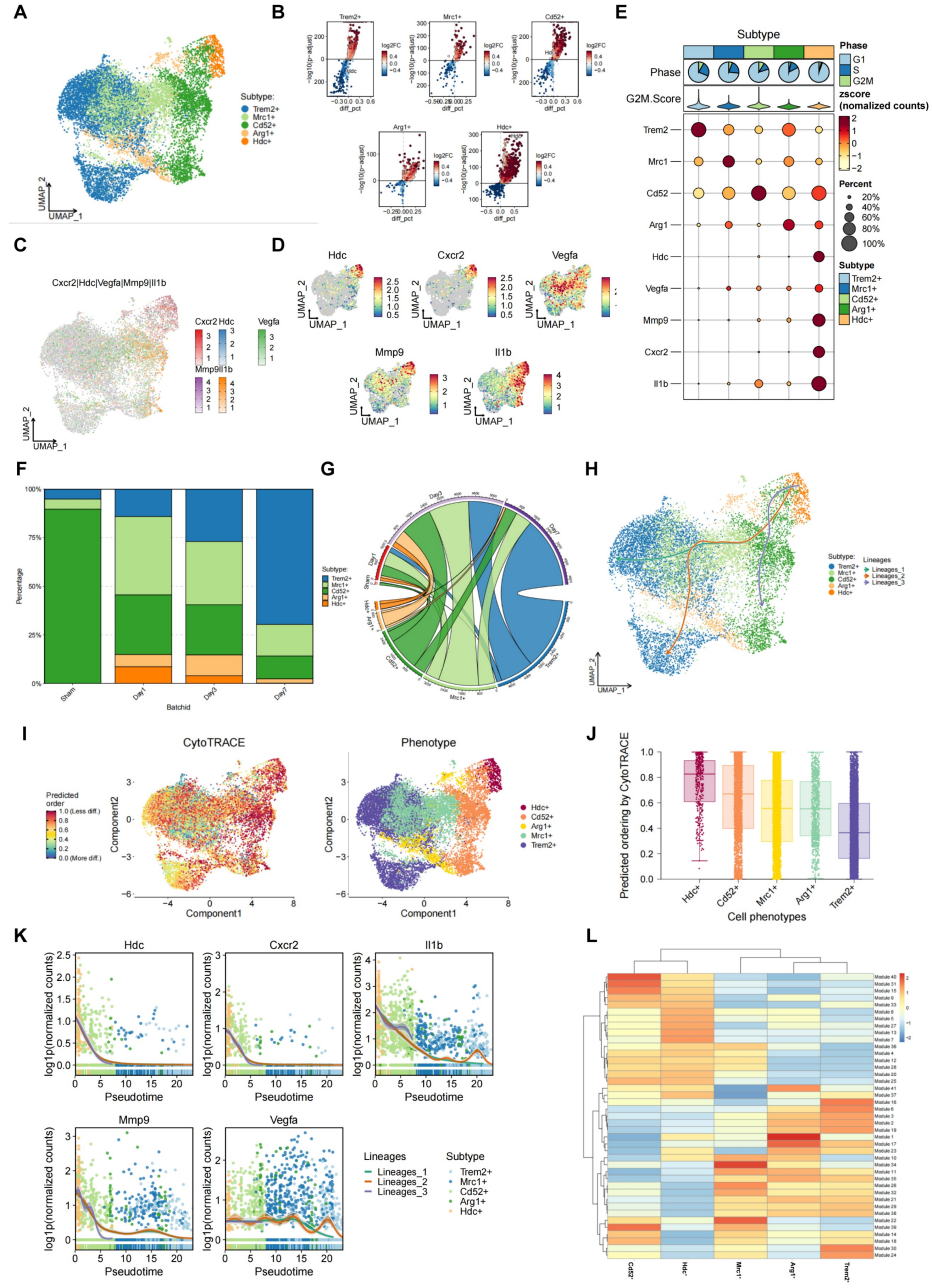
** Single-cell transcriptomics analysis suggests an association between HDC^+^ macrophages and angiogenesis during the process of HLI. (A)** UMAP plot of aggregate cells displaying five macrophage subtypes. **(B)** Volcano plots of differentially expressed genes in each macrophage subtype. **(C)** Total plot showing the expression of Hdc, Cxcr2, Vegfa, Mmp-9and Il1β genes in five macrophage subtypes. **(D)** Feature plots showing the expression of Hdc, Cxcr2, Vegfa, Mmp-9 and Il1β genes in five macrophage subtypes. **(E)** Heat map showing the expression of Hdc, Cxcr2, Vegfa, Mmp-9 and Il1β genes in five macrophage subtypes. **(F)** Bar chart of muscle macrophage cluster proportion at different time after HLI in WT mice. **(G)** Chord diagram of muscle macrophage cluster proportion at different time after HLI in WT mice. **(H)** UMAP plot of differentiation trajectory in each macrophage subtype after HLI in WT mice. **(I)** Plot of the CytoTRACE pseudo-time order for the macrophage subtypes. **(J)** Calculation of CytoTRACE scores of each macrophage cell subtype according to predicted order. **(K)** Dynamic expression of Hdc, Cxcr2, Vegfa, Mmp-9 and Il1β genes along pseudo time. **(L)** WGCNA analysis showing gene modules with similar expression models in five macrophage subtypes.

To further explore the relationship between infiltrating macrophage subsets and angiogenesis during hindlimb ischemia, Single-cell transcription analysis of hindlimb skeletal muscle tissue was performed on post-op day 1 (sham and HLI) and post-op days 3 and 7 (HLI only) utilizing an online Single-cell RNA sequencing database. First, the result of clustering analysis revealed 12 cell clusters including fibro/adipogenic progenitors (FAPs), macrophages, B cells, MuSCs, neutrophils, cycling basal cells, endothelial cells, Schwann cells, NK/T cells, tenocytes erythrocytes, and epithelial cells (Figure S3A). Clusters were categorized based on the expression of established marker genes associated with specific cell types (Figure S3B, C, D). Subsequently, the single-cell profiles corresponding to macrophage clusters were re-clustered, leading to the discovery of five distinct macrophage clusters (Figure 3A, B). Interestingly, a macrophage subset with high level of Hdc expression was identified among five macrophage clusters and these HDC^+^ macrophages also expressed Cxcr2, Il-1β, Mmp-9, and Vegfa at high levels, suggesting their multiple functions in the chemotactic migration, inflammation, and angiogenesis during the development of HLI (Figure 3C, D, E). The identification of the HDC^+^ CXCR2^+^ macrophage subset in mice was provided in the supplementary materials (Figure S3D-H). By analyzing the proportion of macrophages at different times, HDC^+^ macrophages were detected to mainly appear 1-3 days after ischemic injury, suggesting their critical roles in the early stage of HLI (Figure 3F, G). We next performed the CytoTRACE analysis to evaluate the evolutionary dynamics of macrophage subtypes in the process of HLI. The result might reveal the direction of evolution and predicting cell lineage trajectories. Along this trajectory, the expression level of Hdc gene increased in the early stage and decreased rapidly after 3 days (Figure 3H, I, J). Other genes, including Cxcr2, Il-1β, Mmp-9, and Vegfa also exhibited same expression pattern (Figure 3K). In addition, WGCNA data demonstrated a distinct co-expressed gene module with top co-expressed genes including Hdc, Cxcr2, and Mmp-9, indicating these genes have related functions in HLI (Figure 3L). Therefore, these data suggest that HDC^+^ macrophages are likely involved in angiogenesis via the regulation of Cxcr2, Il-1β, Mmp-9, and Vegfa signaling pathways, particular in the early stage of HLI.

### Hdc knockout induced atypical macrophage polarization and down-regulated pro-angiogenic factors expression during HLI by regulating NF-κB and MAPK pathways

**Figure 4:**
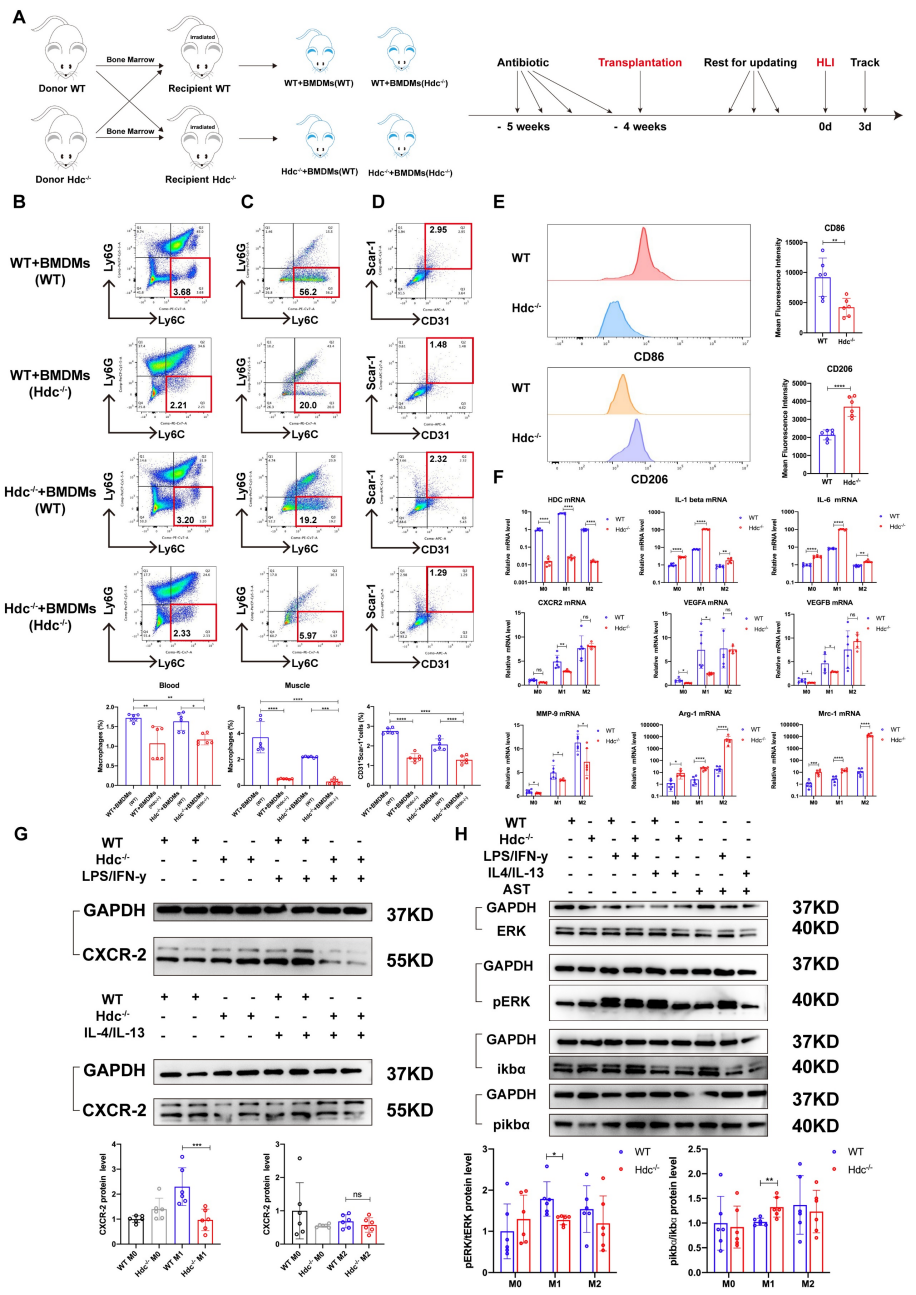
** Hdc knockout induced atypical macrophage polarization and down-regulated pro-angiogenic factors expression during HLI by regulating NF-κB and MAPK pathways. (A)** Scheme showing bone marrow transplantation experiment. **(B)** Representative images and quantification of FACS analysis of the macrophages percentage in the peripheral blood of WT and Hdc^-/-^ mice transplanted with the bone marrow of each other 3 days after HLI (n=6). **(C)** Representative images and quantification of FACS analysis of the macrophages percentage in the muscle tissue of WT and Hdc^-/-^ mice transplanted with the bone marrow of each other 3 days after HLI; (n=6). **(D)** Representative images and quantification of FACS analysis of the CD31^+^Scar-1^+^ endothelial cells percentage in the muscle tissue of WT and Hdc^-/-^ mice transplanted with the bone marrow of each other 3 days after HLI (n=6). **(E)** Representative images and quantification of FACS analysis of the CD86^+^ BMDMs or CD206^+^ BMDMs percentage of WT and Hdc^-/-^ mice (n=6). **(F)** qPCR showing mRNA levels of indicated genes in BMDMs of WT and Hdc^-/-^ mice. BMDMs were treated with LPS/IFN-γ or IL-4/IL-13 for 24 h (n=6). **(G)** Western blot images and quantitative analysis of CXCR2 expression in BMDMs of WT and Hdc^-/-^ mice. BMDMs were treated with LPS/IFN-γ or IL-4/IL-13 for 24 h (n=6). **(H)** Western blot images and quantitative analysis of protein of NF-κB and MAPK pathways expression in BMDMs of WT and Hdc^-/-^ mice. BMDMs were treated with LPS/IFN-γ or IL-4/IL-13 for 24 h; (n=6). For all experiments, error bars represent the mean ± SD. *P < 0.05, **P < 0.01, ***P < 0.001, ****P < 0.0001.

The bone marrow transplantation was performed to investigate the role of HDC^+^ macrophage during angiogenesis using WT and Hdc^-/-^ mice and experiment plan diagram was shown in Figure 4A. FACS data showed a notable decrease in the quantity of CD11b^+^Ly6C^+^ macrophages in the peripheral blood and ischemic muscle of Hdc^-/-^ mice compared to their WT counterparts 3 days after HLI (Figure 4B, C, Figure S4A, B). BMC transplantation from WT mice partially rescued the downregulation of CD11b^+^Ly6C^+^ macrophages in the peripheral blood and ischemic muscle of Hdc^-/-^ mice, albeit not to the WT level (Figure 4B, C, Figure S4A, B). Additionally, FACS data revealed a higher proportion of CD31^+^ endothelial cells in the ischemic muscle of Hdc^-/-^ mice that underwent WT bone marrow transplantation (WT bone marrow contains HDC^+^Ly6C^+^ monocytes/macrophages), indicating the improvement of revascularization (Figure 4D).

To further elucidate the molecular mechanisms by which HDC regulates macrophage differentiation and function, RNA-seq transcriptome sequencing technology was performed on injured lower limb muscles of Hdc^-/-^ and WT mice. Differential gene analysis showed that Cxcr2, Mmp-9, Vegfa were significantly changed after knockout of Hdc (Figure S4C, D). Combining single cell transcriptomics data with RNA-seq data, the key angiogenesis related genes that Hdc may regulate in macrophages included Cxcr2, Il-1β, and Mmp-9 (Figure S4E). Macrophage differentiation model was established using BMDMs isolated from the bone marrow of WT and Hdc^-/-^ mice, and M1-like phenotype and M2-like phenotype were examined respectively by Phase contrast microscope and FACS (Figure S4F). Interestingly, FACS data shows that under M1 polarization conditions, the expression level of CD86 in Hdc^-/-^ BMDMs is lower than that in WT BMDMs, but under M2 polarization conditions, the expression level of CD206 in Hdc^-/-^ BMDMs is higher than that in WT BMDMs (Figure 4E). Furthermore, the results of RT-qPCR and ELISA demonstrated higher levels expression of IL-1β and IL-6 and lower levels expression of Cxcr-2, Vegfa, Vegfb, and Mmp-9 in Hdc^-/-^ BMDMs compared to WT BMDMs (Figure 4F and Figure S4G). In addition, WB result showed decreased expression of Cxcr-2 in Hdc^-/-^ BMDMs compared with WT BMDMs under M1 polarization conditions, indicating a decrease in the chemotactic ability of Hdc^-/-^ BMDMs in the early stage of HLI (Figure 4G).

To explore the intracellular mechanism of HDC/histamine signaling on macrophage function, differential gene description and pathway enrichment analysis were performed for different subpopulations of macrophages in muscle tissue of WT and Hdc^-/-^ mouse after HLI. Among multiple significantly altered signaling pathways, NF-κB and MAPK signaling pathway were screened, indicating a close correlation with HDC/histamine regulation of macrophage function (Figure S4H, S4I). Consequently, the expression levels of ERK, pERK, ikbα, and pikbα were measured in WT and Hdc^-/-^ BMDMs. Western blot data demonstrated decreased phosphorylation of ERK, but increased phosphorylation of ikbα in Hdc^-/-^ BMDMs compared to WT BMDMs (Figure 4H). Collectively, these findings indicate that the disruption of HDC/histamine signal in Hdc^-/-^ mice may induce the atypical polarization of macrophages, enhance inflammatory response, and decrease angiogenesis factors via the regulation of MAPK, NF-κB, and ERK pathways during the process of HLI.

### HDC^+^ macrophages combine to endothelial cells via CXCR2-CXCL2 loop and promote angiogenesis mediated by VEGF, MMP-9, and IL-1β

**Figure 5:**
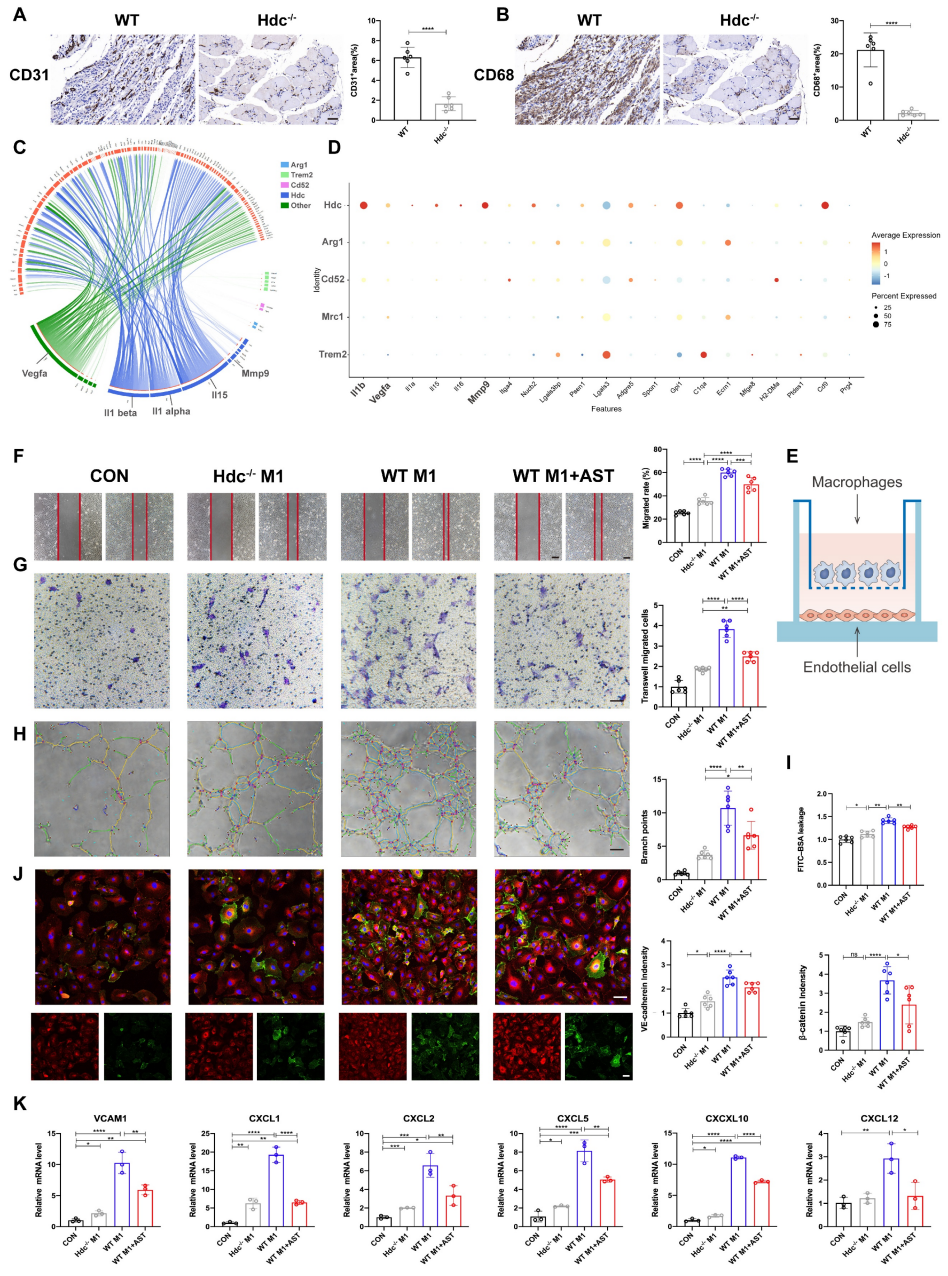
** HDC^+^ macrophages combine to endothelial cells via CXCR2-CXCL2 loop and promote angiogenesis mediated by VEGF, MMP-9, and IL-1β. (A)** Representative images and quantitative analysis of Anti-CD31 immunohistochemistry staining of injured gastrocnemius muscle from Hdc^-/-^ and WT mice at day 3 post-injury in ischemic muscles; Scale bar, 50µm; (n=6). **(B)** Representative images and quantitative analysis of Anti-CD68 immunohistochemistry staining of injured gastrocnemius muscle from Hdc^-/-^ and WT mice at day 3 post-injury in ischemic muscles; Scale bar, 50µm; (n=6). **(C)** Predict ligands from different macrophage subpopulations that regulate endothelial cell target genes. Ligands are annotated by the cell type that expresses them. Edges are scaled by the inferred regulatory potential of the interaction. **(D)** Heatmap of ligands that regulate respective target genes of endothelial cells from different populations of macrophages. **(E)** Scheme showing HUVECs co-cultured with WT BMDMs and Hdc^-/-^ BMDMs.** (F)** Representative images and quantification of scratch wound healing assay of HUVECs co-cultured with WT BMDMs and Hdc^-/-^ BMDMs. HUVECs were pretreated with 10μM H_2_O_2_ and 1μm AST; Scale bars, 50µm; (n=6). **(G)** Representative images and quantification of transwell assays of HUVECs co-cultured with WT BMDMs and Hdc^-/-^ BMDMs. HUVECs were pretreated with 10μM H_2_O_2_ and 1μm AST; Scale bars, 50µm; (n=6). **(H)** Representative images and quantification of tube formation assay of HUVECs co-cultured with WT BMDMs and Hdc^-/-^BMDMs. HUVECs were pretreated with 10μM H_2_O_2_ and 1μm AST; Scale bars, 50µm; (n=6). **(I)** Endothelial monolayer permeability was evaluated by FITC-BSA leakage assay; (n=6). **(J)** Representative images and quantitative analysis of VE-cadherin (red) and β-catenin (green); Scale bars, 50µm; (n=6). **(K)** qPCR showing mRNA levels of indicated genes in co-cultured with WT BMDMs and Hdc^-/-^BMDMs. HUVECs were pretreated with 10μM H_2_O_2_ and 1μm AST; (n=3). For all experiments, error bars represent the mean ± SD.*P < 0.05, **P < 0.01, ***P < 0.001, ****P < 0.0001.

The findings from immunohistochemical staining using anti-CD31 and anti-CD68 antibodies indicated a reduction in the quantity of blood vessels within the damaged muscle tissue of Hdc^-/-^ mice in comparison to WT mice three days post hindlimb ischemia, accompanied by a decrease in macrophage infiltration, suggesting that angiogenesis is related to the dysfunction of macrophages (Figure 5A, B). Consequently, NicheNet algorithm was conducted to get a systematic understanding for the cross-talk between macrophages and endothelial cells. The results of association analysis displayed that HDC^+^CXCR2^+^ macrophage subset expressed more genes regulating endothelial cells and had stronger interactions with endothelial cells than other populations of macrophage subsets (Figure 5C). Heatmap showed that HDC^+^CXCR2^+^ macrophages and endothelial cells interact mainly through IL-1β, Vegfa, and Mmp-9, which less expressed in other macrophage subsets (Figure 5D). Cell chat and associated heat maps showed a higher degree of interaction between HDC^+^CXCR2^+^ macrophages and endothelial cells via VEGF signaling compared to other macrophage subsets (Figure S5A, B). Furthermore, BMDMs from WT and Hdc^-/-^ mice were co-cultured with HUVECs to explore the effect of macrophage secreted factors on endothelial function (Figure 5E). The integration results of Scratch wound healing assays (Figure 5F), transwell assays (Figure 5G) and tube formation assays (Figure 5H) showed that Hdc^-/-^ BMDMs treated HUVECs underwent significantly decreased migration compared with WT BMDMs treated HUVECs. Endothelial monolayer permeability test (Figure 5I) and immunofluorescent staining (Figure 5J) showed that HUVECs treated with WT BMDMs were more closely connected than HUVECs treated with Hdc^-/-^ BMDMs, which may be due to the release of more inflammatory factors by Hdc^-/-^ BMDMs. Moreover, the findings from both the cell viability assay and tube formation assay indicate that brief exposure to M1 macrophages promotes vascularization, whereas prolonged exposure leads to impaired vessel formation. Hdc^-/-^ M1 macrophages have a decreased pro-angiogenic effect s during the early stage of HLI and exacerbate vascular degeneration in the later stages (Figure S5C, D). RT-qPCR data demonstrated that Hdc^-/-^ BMDMs treated HUVECs had a decreased gene expression of CXCLs compared to WT BMDMs, suggesting a decreased capacity of migrating in HUVECs (Figure 5K). These data indicate that CXCR2-CXCL2-mediated chemotaxis can increase recruitment and infiltration of immune cells after HLI. Endothelial cells treated with Hdc^-/-^ BMDMs shave less ability to recruit macrophages, which are unable to form a microenvironment that promotes angiogenesis.

### Histamine promotes endothelial cell migration and tube formation by activating H_1_R and CXCL/PI3K/AKT signaling pathway

**Figure 6:**
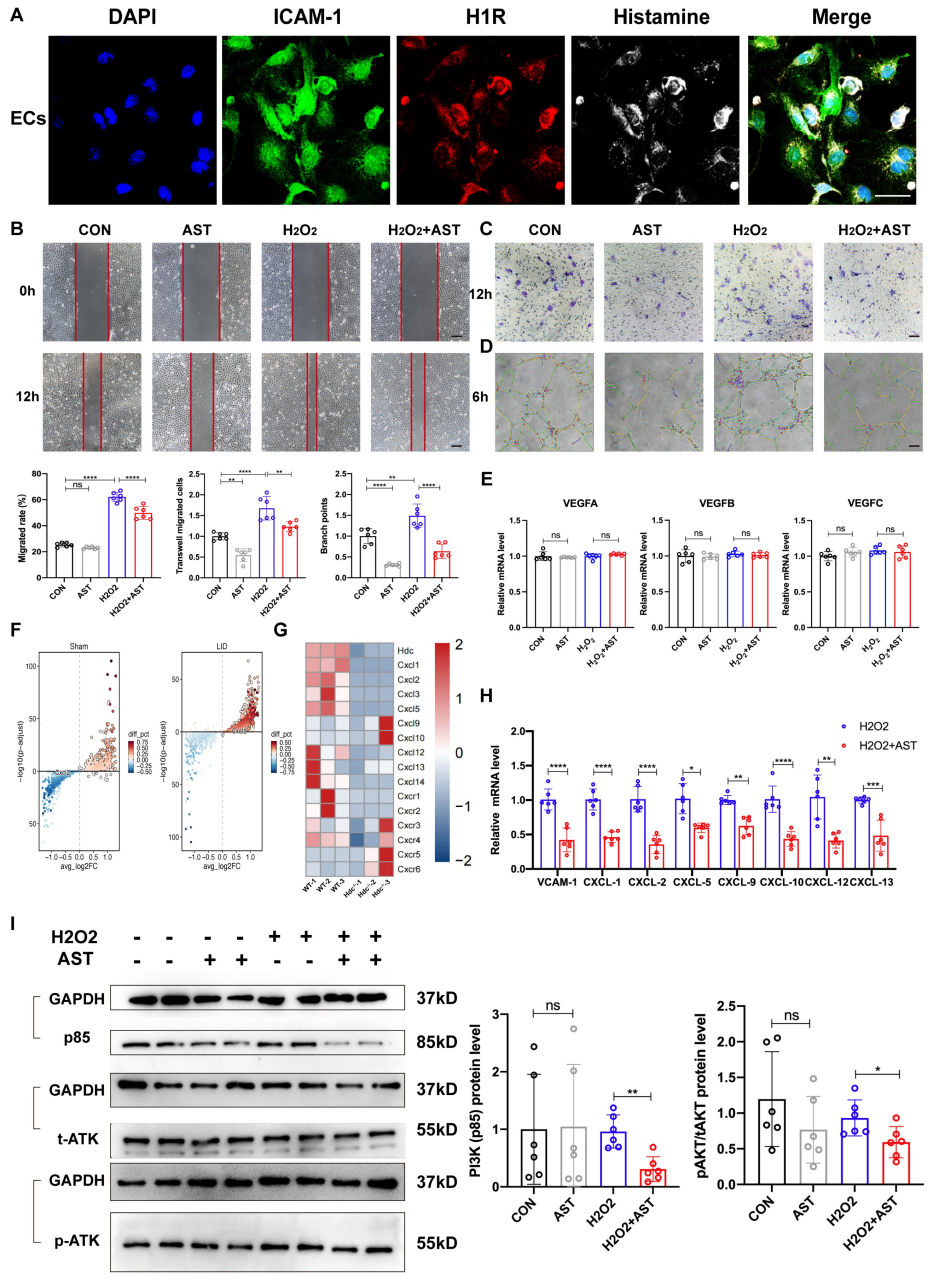
** Histamine promotes endothelial cell migration and tube formation by activating H_1_R and CXCL/PI3K/AKT signaling pathway. (A)** Representative images of ICAM-1(green), H_1_R (red), histamine (white) and DAPI (blue) immunostainings of HUVECs; scale bar, 20µm. **(B)** Representative images and quantification of scratch wound healing assay of HUVECs cultured in conditioned medium with or without 1μM AST. HUVECs were pretreated with 10μM H_2_O_2_. Scale bars, 50µm; (n=6). **(C)** Representative images and quantification of transwell assays of HUVECs cultured in conditioned medium with or without 1μM AST. HUVECs were pretreated with 10μM H_2_O_2_. Scale bars, 50µm; (n=6). **(D)** Representative images and quantification of tube formation assay of HUVECs cultured in conditioned medium with or without 1μM AST. HUVECs were pretreated with 10μM H_2_O_2_. Scale bars, 50µm; (n=6). **(E)** qPCR showing mRNA levels of indicated genes in HUVECs treated with 1μm AST for 24 h. HUVECs were pretreated with 10μM H_2_O_2_; (n=6). **(F)** Volcano plot of the expression difference of CXCL2 in ischemia muscle tissue of WT mice before and after HLI. Red dots indicate transcripts that were increased (padj< 0.05, Log2 fold change > 1), whereas blue dots indicate decreased transcripts (padj< 0.05, Log2 fold change < 1). **(G)** Heat map of the expression difference between ischemia muscle tissue of WT mice and Hdc^-/-^ mice 3 days after HLI. **(H)** qPCR showing mRNA levels of indicated genes in HUVECs treated with 1μm AST for 24 h. HUVECs were pretreated with 10μM H_2_O_2_; (n=6). **(I)** Western blot images of protein of PI3K/AKT pathway expression in HUVECs treated with 1μm AST for 24 h. HUVECs were pretreated with 10μM H_2_O_2_.HUVECs were pretreated with 10μM H_2_O_2_; (n=6). For all experiments, error bars represent the mean ± SD. *P < 0.05, **P < 0.01, ***P < 0.001, ****P < 0.0001.

Except of secreting pro-angiogenic factors, HDC^+^ macrophages directly activate endothelial receptors via histamine release. The immunofluorescence staining result demonstrated the expression of H_1_R and H_2_R in human umbilical vein endothelial cells (HUVECs) (Figure 6A, S6A). Astemizole (AST, a selective H_1_R antagonist) and Cimetidine (CIM, a selective H_2_R antagonist) were used to treat HUVECs to explore related molecular mechanisms. First, Cell viability assay demonstrated that H_1_R antagonist AST, not H_2_R antagonist CIM treatment could reduce HUVECs viability (Figure S6B). Second, the result of a scratch wound healing assay confirmed that AST treatment significantly decreased HUVECs migration compared with controls (Figure 6B). Transwell assays data also validated that the migration of HUVECs was decreased upon the blockage of H_1_R by AST (Figure 6C). In addition, a significant decrease of tube formation was detected when HUVECs were treated with AST (Figure 6D). However, CIM treatment has poor effect in the above experiments (Figure S6C, D, E).

We used RT-qPCR to examine whether blocking histamine receptors could reduce VEGF secretion in endothelial cells and found no significant change (Figure 6E). The integrated analysis of single-cell transcriptomic data in endothelial cells (Figure 6F) and RNA sequence data in the ischemic limb muscle (Figure 6G) revealed the significant changes of chemokine ligands (CXCLs) in WT and Hdc^-/-^ mice 3 days after HLI. RT-qPCR data also confirmed significant down-regulation of vascular cellular adhesion molecule-1 (VCAM-1) and CXCLs in cultured endothelial cells with AST treatment (Figure 6H). The phosphatidylinositol 3-kinase (PI3K)/AKT signaling pathway is an intracellular signal that regulates a variety of cellular functions in response to extracellular signals. Furthermore, Western Blot data revealed that blocking H_1_R results in decreased protein levels of PI3K (P85) and inhibited phosphorylation of AKT in endothelial cell with AST treatment (Figure 6I). These results collectively indicate that histamine may enhance the expression of VCAM-1 and CXCLs, promote endothelial cell migration and tube formation through the activation of H_1_R-PI3K-AKT signaling pathway.

### Blocking H_1_R receptors is not conducive to the repair of lower limb ischemic injury in mice and patients

**Figure 7:**
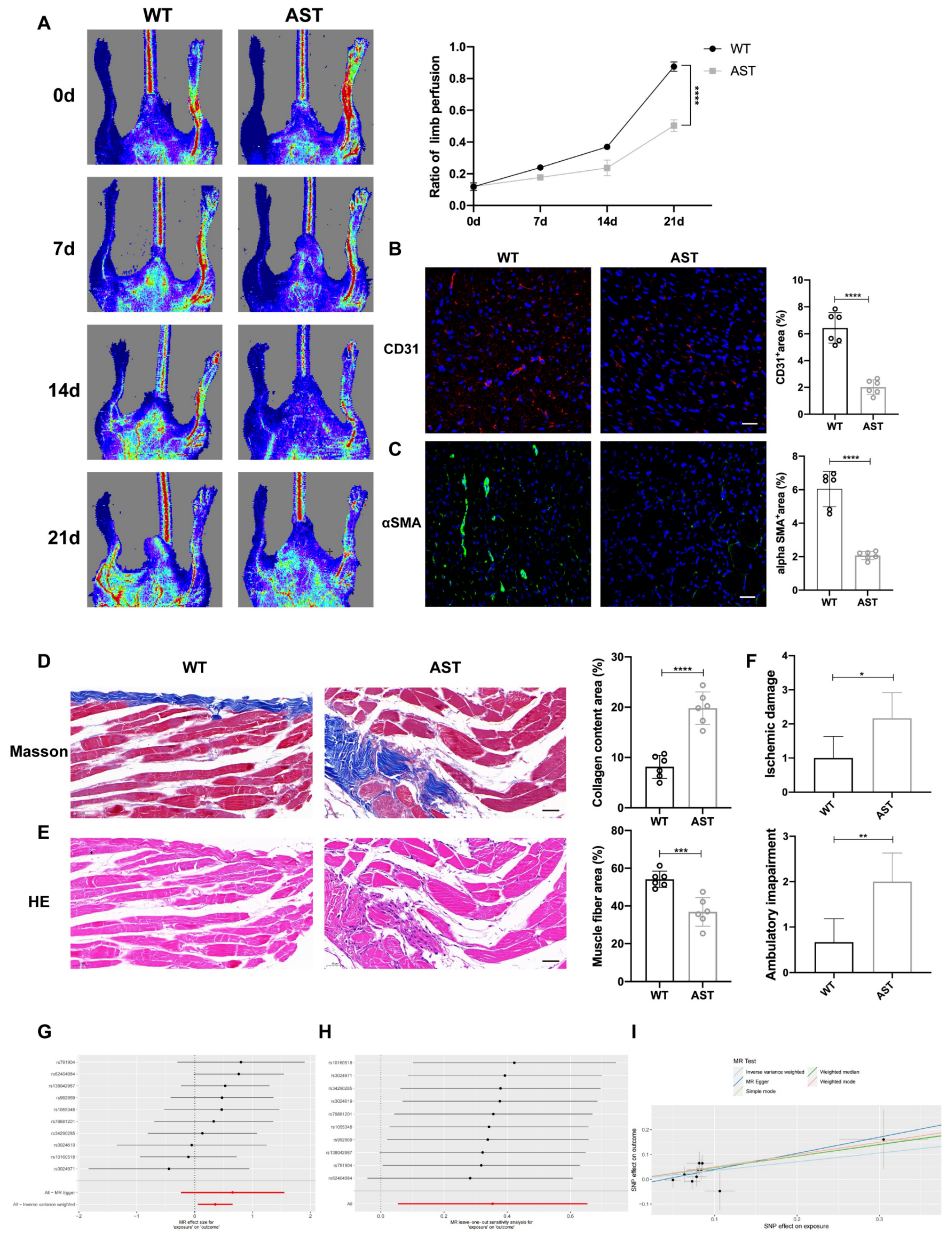
** Blocking H_1_R receptors is not conducive to the repair of lower limb ischemic injury in mice and patients. (A)** Representative images and quantification of hindlimb blood perfusion in WT and AST-injected mice using laser Doppler imaging at 0, 7, 14 and 21 days after femoral ligation (n=6). Red, green, and blue indicated the fastest, intermediate, and slowest blood flow velocities, respectively. **(B)** Representative images of CD31 (red), and DAPI (blue) immunostainings and quantification of CD31^+^ ECs in muscles at 3 days (n=6); scale bar, 50µm. **(C)** Representative images of αSMA (green), and DAPI (blue) immunostainings and quantification of αSMA^+^ cells in muscles at 3 days (n=6); scale bar, 50µm. **(D)** Representative images and quantitative analysis of Masson's staining of injured gastrocnemius muscle from WT and AST-injected mice at day 21 post-injury in ischemic muscles (n=6); Scale bar, 50µm. **(E)** Representative images and quantitative analysis of H&E staining of injured gastrocnemius muscle from WT and AST-injected mice at day 21 post-injury in ischemic muscles (n=6); Scale bar, 50µm. **(F)** Ischemic damage scores, ambulatory impairment scores in WT and AST-injected mice (n=6). **(G)** Forest plots of genetic association between the use of histamine receptor antagonists and the prognosis of PAD. **(H)** Scatter plot of genetic association between the use of histamine receptor antagonists and the prognosis of PAD. **(I)** Forest plots that removed single impact points. For all experiments, error bars represent the mean ± SD.*P < 0.05, **P < 0.01, ***P < 0.001, ****P < 0.0001.

To further investigate the role of blocking histamine receptor H_1_R *in vivo*, we administered 2 mg/(kg·d) AST intraperitoneally to WT mice for three consecutive days and established a hindlimb ischemia model. Laser Doppler imaging showed that, compared to WT control mice, the recovery of blood flow in the ischemic limbs of mice receiving intraperitoneal injections of AST was significantly delayed (Figure 7A). Immunofluorescence staining with anti-CD31 and anti-α-SMA^+^ revealed that on day 3 after HLI, the number of CD31^+^ endothelial cells and α-SMA^+^ cells in the ischemic muscle of AST-injected mice was significantly reduced (Figure 7B, C). Compared to WT control mice, the vascular density in the affected limbs was lower after AST treatment. Masson staining and hematoxylin and eosin (H&E) staining images showed that 21 days after HLI, AST-injected mice exhibited severe fibrosis and muscle necrosis compared to WT control mice (Figure 7D, E). Furthermore, the limb function scores for ischemic damage and motor impairment were evaluated. The results indicated that AST-injected mice had higher scores in both categories, suggesting a restricted capacity for functional regeneration in ischemic limbs by day 21 (Figure 7F). In summary, these findings indicate that blocking histamine H_1_R exerts an inhibitory effect on revascularization and skeletal muscle regeneration following HLI.

Histamine receptor antagonists are widely used in clinical practice, especially in anti-allergic reactions (H_1_R antagonist) and gastric acid secretion (H_2_R antagonist). However, the effect of histamine/HR signal on angiogenesis in panvascular diseases is rarely studied. Peripheral artery disease (PAD) data from online data base was used to conduct a Mendelian randomization study. Histamine receptor antagonists were used as exposure factors, and sequelae of lower limb injury were used as outcome indicators. The results of Forest plots and scatter plots showed that the use of histamine receptor antagonists had a significant damaging effect on the prognosis of PAD, and the difference was statistically significant (Figure 7G, H, Table S5. No high-impact points were found in the leave-one-out analysis (Figure 7I, Table S6, S7). These findings suggest that histamine receptor antagonists may negatively impact angiogenesis and tissue repair in peripheral artery disease, highlighting the need for further investigation into their potential effects and careful consideration in clinical applications.

### Histamine-delivering Hydrogel promotes skeletal muscle regeneration in Hdc^-/-^ mice after HLI by regulating angiogenesis and inflammatory disorders

Previous studies have shown that intraperitoneal or subcutaneous injection of exogenous histamine can alleviate myocardial injury caused by acute myocardial infarction or chemotherapy drugs in mice 17, 30. However, considering the half-life of histamine and the toxic side effects caused by excessive input of histamine, we plan to develop a new material that can sustainably release histamine, regulate angiogenesis and inflammatory responses, and promote skeletal muscle injury repair.

**Figure 8:**
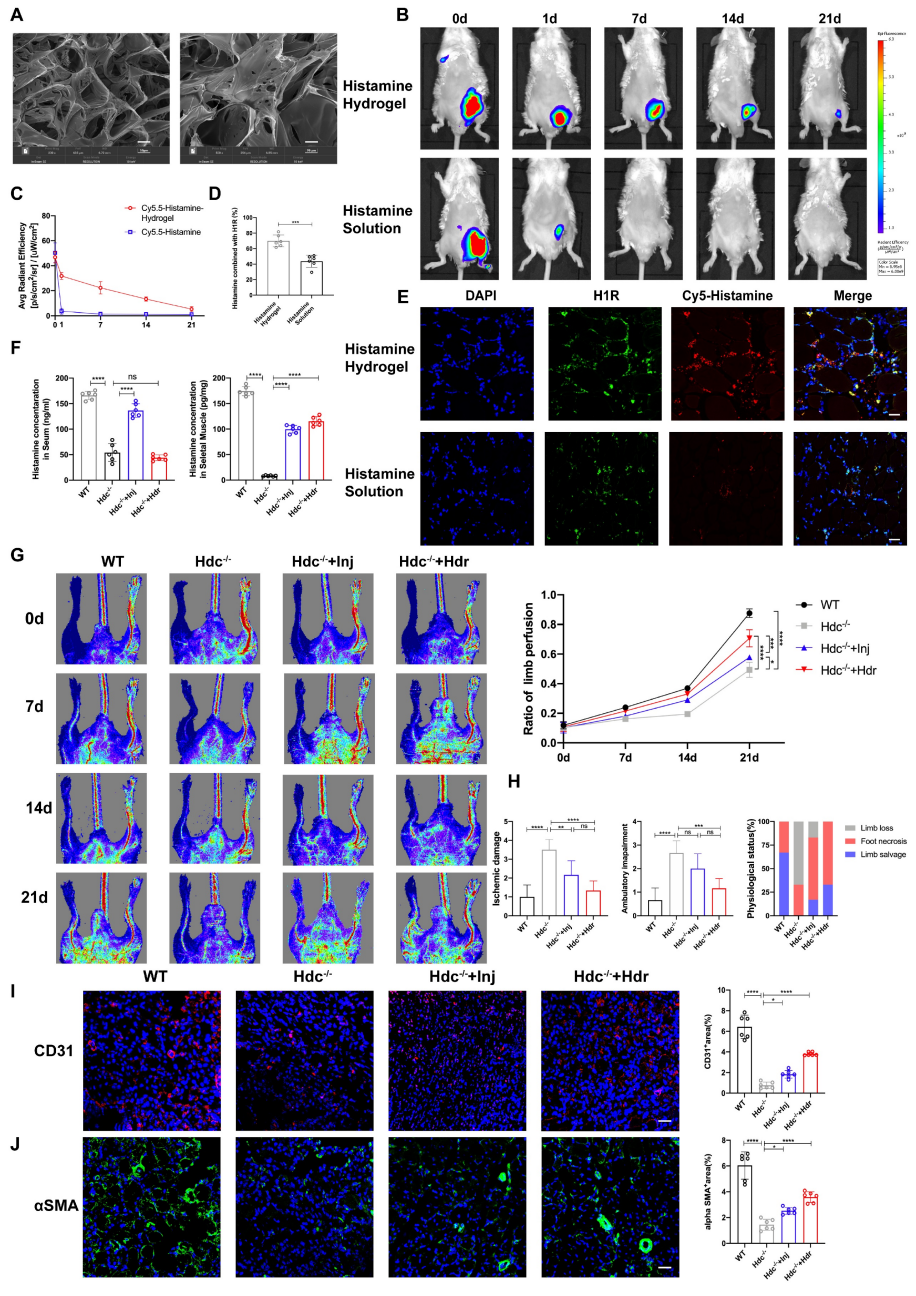
** Histamine-delivering Hydrogel promotes skeletal muscle regeneration in Hdc^-/-^ mice after HLI by regulating angiogenesis and inflammatory disorders. (A)** SEM image of HA-DA@histamine hydrogel; left: Scale bar, 50µm; right: Scale bar, 20µm. **(B)** Measuring the retention of injected naked Cy5.5-Histamine or Cy5.5-labeled histamine in HA-DA hydrogel group via tracking fluorescence signal by *in vivo* imaging system.** (C)** Quantitative analysis of fluorescence signals in the mice limb (n=6). **(D)** and **(E)** Representative images of H_1_R (green), Cy5.5-Histamine (red), and DAPI (blue) immunostainings **(E)** and quantification **(D)** of binding ratio of H_1_R and histamine at 3 days (n=6); scale bar, 20µm. **(F)** Histamine in the serum and in the ischemia muscle tissue of HLI murine model at 7 days after HLI surgery was determined by Elisa kit (n=6). **(G)** Representative images and quantification of hindlimb blood perfusion in each group using laser Doppler imaging at 0, 7, 14 and 21 days after femoral ligation (n=6). Red, green, and blue indicated the fastest, intermediate, and slowest blood flow velocities, respectively. **(H)** Ischemic damage scores, ambulatory impairment scores, and percentage distribution analysis of limb salvage, foot necrosis, and limb loss in each group (n=6). **(I)** Representative images of CD31 (red), and DAPI (blue) immunostainings and quantification of CD31^+^ ECs in muscles at 3 days (n=6); scale bar, 50µm. **(J)** Representative images of αSMA (green), and DAPI (blue) immunostainings and quantification of αSMA^+^ cells in muscles at 3 days (n=6); scale bar, 50µm. For all experiments, error bars represent the mean ± SD.*P < 0.05, **P < 0.01, ***P < 0.001, ****P < 0.0001.

In the current study, we designed an injectable Dopamine (DA)-cross linked hyaluronan (HA) hydrogel with delivery of histamine Figure S7A). The hydrogel sample's morphology was analyzed using a Scanning Electron Microscope (SEM) (Figure 8A). The rheological properties of the HA-DA@histamine hydrogel were examined to analyze the correlation between viscosity and shear rate, providing insights into the injectability of the hydrogel (Figure S7B). The hydrogel's swelling behavior in phosphate-buffered saline (PBS) at 37°C was monitored. The hydrogel sample exhibited rapid swelling within the initial 2-hour period upon immersion in PBS (Figure S7C). The degradation rate of HA-DA@histamine hydrogel was 68% on day 14 (Figure S7D). The rate of histamine release from the hydrogel at 37°C was assessed using a BCA assay, which indicated that histamine could be efficiently loaded into the hydrogel and maintain a sustained release profile for up to 7 days (45%) and 14 days (65%) at 37°C (Figure S7E).

In order to ascertain the potential of hydrogel in enhancing the retention and stability of histamine in an *in vivo* setting, Cy5-labeled histamine incorporated with hydrogel (Histamine/Hydrogel group) or natural histamine (Histamine group) were administered intramuscularly to WT mice. At the predetermined time points, the results of *in vivo* imaging system showed that the fluorescence intensities in Histamine/Hydrogel group gradually decayed during 21 days, but rapidly decreased in one day in Histamine group (Figure 8B, C). Immunofluorescent detection confirmed that histamine was released from the hydrogel and bound to H_1_R on vascular endothelium (Figure 8D, E). Elisa data showed that local injection of histamine sustained-release gel can increase histamine concentration in the muscle tissue, but very little in the serum (Figure 8F).

To investigate the therapeutic efficacy of HA-DA@histamine Hydrogel on HLI, the hydrogel was administered into the limbs of Hdc^-/-^ mice post-HLI, with WT mice or Hdc^-/-^ mice receiving intraperitoneal injections of histamine serving as control groups. The analysis of Laser Doppler imaging data revealed a significant enhancement in the ratio of blood perfusion following the injection of HA-DA@histamine Hydrogel in Hdc^-/-^ mice, as compared to the other three groups (Figure 8G). Hdc^-/-^ mice that received hydrogel injections exhibited a reduced incidence of necrotic toes following hindlimb ischemia (HLI) (Figure S7F). Furthermore, the results of Masson and H&E staining confirmed that mice in HA-DA@histamine Hydrogel group showed less inflammatory response and fibrosis compared with mice in control groups (Figure S7G, H). The limb function scores pertaining to ischemic damage and ambulatory impairment demonstrated that HA-DA@histamine Hydrogel effectively enhanced functional recovery in the lower limbs of mice with hindlimb ischemia (Figure 8H). In addition, immunofluorescent analysis revealed a significant increase in the quantities of CD31^+^ cells and αSMA^+^ cells within the HA-DA@histamine Hydrogel group when compared to the Hdc^-/-^ control group (Figure 8I, J). FACS data confirmed more CD31^+^Sca-1^+^arteriole cells in HA-DA@histamine Hydrogel group than in Hdc^-/-^ control group (Figure S7I). In summary, the use of the histamine-releasing hydrogel provides a moderate and stable compensation for histamine deficiency in hindlimb ischemic injury. This approach promotes angiogenesis and tissue repair in HLI mice.

## Discussion

**Figure 9:**
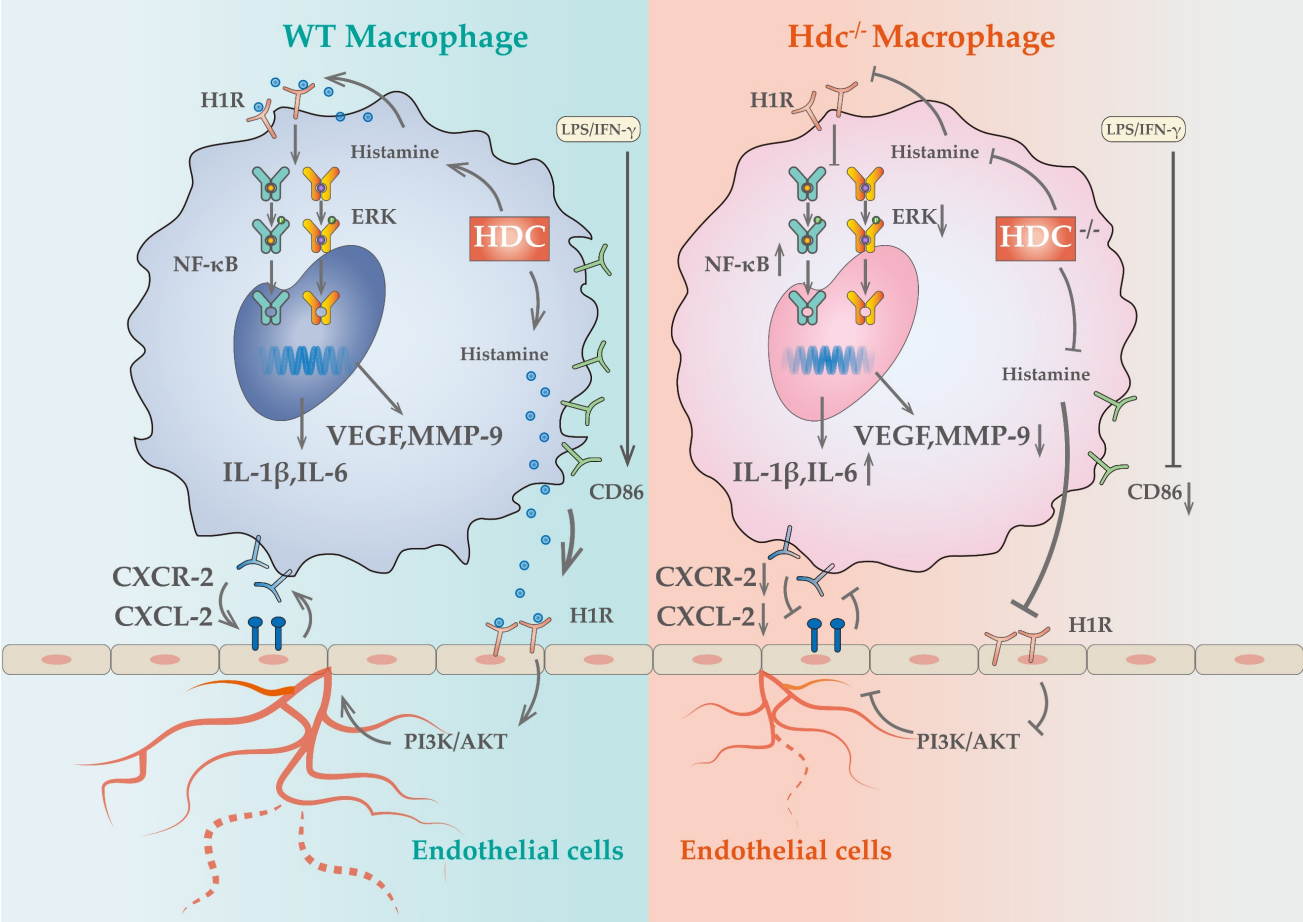
Schematic showing the differences of functions and mechanisms between WT BMDMs and Hdc^-/-^ BMDMs.

Panvascular diseases encompass a range of vascular diseases characterized by atherosclerosis as a predominant pathological feature, primarily impacting vital organs including the heart, brain, kidneys, and extremities, clinically manifested as coronary artery disease, ischemic stroke, and peripheral artery disease (1, 31. These patients require comprehensive multi-specialty treatment and panvascular revascularization 1, 32. Early revascularization of ischemic tissue is crucial to promote targeted organ damage repair 33. However, there remains a significant gap in knowledge regarding the underlying mechanisms of angiogenesis in ischemic tissue. In the present study, we uncovered a cluster of macrophages with high level expression of HDC in ischemic tissue that closely related to angiogenesis and skeletal muscle regeneration through combined scRNA-seq and fluorescent tracing technologies. Histamine deficiency in Hdc knockout mice (Hdc^-/-^) not only impaired macrophage differentiation and function, but also repressed the migration and tube-formation of endothelial cells, resulting in delayed angiogenesis and muscle regeneration (Figure 9). Targeting the lack of expression of HDC in skeletal muscle and endothelial cells and the shortage of endogenous histamine in ischemic injury tissue, we designed an injectable hydrogel for sustained release of histamine to supplement exogenous histamine to damaged tissues. The outcomes of both *in vivo* and *in vitro* experiments proved that histamine-releasing hydrogel could create a favorable microenvironment promoting re-vascularization and skeletal muscle regeneration after HLI.

Macrophages play key roles in phases of the response to injury including inflammation, angiogenesis, regeneration, and fibrosis 34. It has been well documented that bone marrow-derived monocytes can be activated and differentiated into classically activated macrophages (M1) and alternative activated macrophages (M2) in response to different inflammatory stimuli 35. Based on the development of single-cell omics technology and lineage tracing technology, the heterogeneity of macrophages has been further demonstrated, posing a challenge to the dichotomy classification of M1/M2 macrophages 36. Recent studies identified several novel macrophage subsets with more specific functions in tumor, myocardial infarction, and lower limb ischemic microenvironment by dimensionality reduction analysis and reclustering 37-39. However, there is controversy in previous studies regarding the impact of macrophages on angiogenesis. For instance, studies have indicated that the secretion of vascular endothelial growth factor (VEGF) from M1 macrophages plays a crucial role in promoting angiogenesis during the initial phases of wound healing in murine models 40, while chronic inflammation caused by the persistent presence of pro-inflammatory (M1) macrophages leads to impaired angiogenesis 41. There are also studies showed that the effect of M1 macrophages on blood vessels varies in time, with its short-term presence (1 day) promoting angiogenesis, but long-term presence (3 days) leading to vascular degeneration 12. In the present investigation, utilizing Hdc-GFP transgenic mice, we provided solid evidence that bone marrow-derived HDC^+^ monocytes/macrophages could be recruited to the ischemic skeletal muscle in the early stage of HLI and regulated angiogenesis. Furthermore, through deep mining of the data of scRNA-seq and spatial transcriptomics, we have identified a cluster of HDC^+^ macrophage subset with high levels gene expression of Cxcr2, Il-1β, Vegfa and Mmp-9 in the process of HLI. The result of lineage trajectories of Hdc and angiogenesis genes showed the similar spatial and temporal distribution of these genes. In addition, *in vitro* macrophage differentiation model was established using the bone marrow of Hdc^-/-^ and WT mice. The results confirmed that dysfunction of HDC in macrophage not only hinders histamine production, but also leads to abnormal differentiation of macrophages, decreased the expression of Vegfa, Mmp-9 and increased the expression of pro-inflammatory cytokines, which may be related to the abnormal activation of MAPK and NF-κB pathways. Therefore, the results of this study highlight that HDC^+^CXCR2^+^ macrophages were closely related to inflammation and angiogenesis in HLI.

The crosstalk between macrophages and endothelial cells is a critical factor in regulating vascular remodeling following ischemic injury 20, 42. Recent data showed that macrophages of distinct phenotypes enhanced angiogenesis by differentially affecting endothelial cell behavior 12, but it is unknown that which genes and pathways are responsible for these effects. The NicheNet algorithm was used in this study to predict ligands of different subtypes of macrophages and their communication with endothelial cell receptors. The results revealed that ligands from HDC^+^ macrophages have the greatest potential to reprogram endothelial cells via ligand-receptor interactions in the early stage of HLI. Among them, histamine/HRs signals occupied very important position. Previous study demonstrated that the activation of histamine/H_3_R/PI3K/AKT signaling pathway involved in the proliferation and maturation of satellite cells in murine muscle regeneration after HLI 18. Histamine H_1_ receptors are primarily situated on the surface of vascular endothelial cells and are essential in regulating vascular dilatation, vascular permeability, and blood pressure 43. Despite the widespread clinical use of H_1_R antagonists, potential side effects including gastrointestinal diseases, arrhythmia, and cardiotoxicity have been reported 44-46. Nevertheless, there is a paucity of research examining the role and mechanism of H_1_R antagonists in angiogenesis. In this study, H_1_R has been identified to be highly expressed in endothelial cells. H_1_R antagonist AST was used to block endothelial cell H_1_R and demonstrated that histamine could promote endothelial cell migration and tubular formation, which mechanism is mainly realized through the H_1_R/CXCL/PI3K/AKT pathway. Mendelian studies based on animal experiments and clinical data have demonstrated that blocking histamine receptors may adversely affect the prognosis of lower limb arterial disease. These findings provide novel insights and evidence for the precise clinical application of histamine receptor antagonists. Given the widespread use of these antagonists in allergic reactions and gastric acid regulation, their potential impact on vascular repair warrants further investigation. Future studies should explore the underlying mechanisms and assess whether alternative therapeutic strategies could mitigate these effects while maintaining their clinical benefits. Previous studies have shown that histamine-deficient mice have abnormal macrophage infiltration and delayed regression after myocardial infarction 30. In this study, we not only observed similar HDC^+^ macrophage mobilization abnormalities, but further demonstrated their atypical macrophage differentiation and reduced expression of pro-angiogenic factors in the early stage of HLI. Furthermore, high level expression of CXCL2 was examined in endothelial cells in response to histamine stimulation and high-level expression of CXCR2 was identified specifically in HDC^+^ macrophages, suggesting that endothelial injury can recruit large number of bone marrow-derived HDC^+^ macrophages through CXCR2-CXCL2 chemotactic axis in HLI. However, knockout of HDC in macrophages inhibits endogenous histamine production and VEGF expression, blocking "the positive feedback loop" between macrophages and endothelial cells. Overall, the results of this study reveal a previously unidentified signaling pathway between HDC^+^ macrophages and endothelial cells that influences the transcriptional phenotypes of both cell types and regulates angiogenesis in HLI.

The effect of macrophages on angiogenesis is a systematic biological process that requires the coordination and integration of a complex set of secretory ligands in space and time 47. Served as forerunners that create a conducive microenvironment for angiogenesis prior to the formation of blood vessels, differentiation regulation of macrophages is very important 48. Numerous studies have demonstrated the capacity of histamine to modulate cell differentiation. Previous study demonstrated that histamine deficiency greatly enhances the proliferation of CD11b^+^Ly6G^+^ tumor associated neutrophils and increased angiogenesis, leading to the development of inflammatory associated colorectal cancer in Hdc^-/-^ mice 17. Recent studies have elucidated the significant involvement of the HDC/histamine signal in the regulation of the transformation of bone marrow-derived macrophages into cardiac myofibroblasts via a HR/KLF5 dependent signaling pathway in acute myocardial infarction mice 49. In infectious diseases, Hdc^-/-^ mice demonstrate impaired macrophage function and differentiation, resulting in increased susceptibility to bacterial infections 50. The regulation of macrophage differentiation by histamine signaling plays a critical role in the maintenance of gastric homeostasis and prevention of bacterial overgrowth 51. Additionally, the population of CD11b^+^Gr-1^+^ immature myeloid cells (IMCs) in the bone marrow, spleen, and peripheral blood of Hdc^-/-^ mice exhibited a significant increase, while the differentiation of CD11b^+^Ly6C^high^ M_1_ macrophages was notably inhibited 30. HDC-expression decreased during the differentiation and maturation of macrophages from monocytes induced by granulocyte-macrophage colony-stimulating factor (GM-CSF) or macrophage colony-stimulating factor (M-CSF) 17. These results suggested that histamine regulates not only the proliferation of monocytes, but also the differentiation and function of macrophages. Prior research has indicated that mast cells are typically considered the primary source of histamine following tissue damage 52. However, our investigation utilizing Hdc-GFP mice revealed that CD11b^+^Gr-1^+^ myeloid cells are the predominant HDC-expressing cells in inflammatory-related tumorigenesis 17, myocardial infarction 30, and lower limb ischemia 18, potentially functioning as progenitors for mature neutrophils and monocytes. Mast cells, on the other hand, serve as transient repositories for histamine and play a role in controlling its release 53. Furthermore, this study on the evolutionary dynamics of macrophage subtypes during HLI unveiled that HDC^+^ macrophages are early in the sequence and deletion of HDC would induce abnormal macrophage differentiation, thus leading to the impairment of the function of promoting angiogenesis. Based on previous research findings and the results of this study, HDC is proposed to be the central driver of macrophages in the angiogenesis of HLI and other panvascular diseases, and is crucial in regulating the differentiation and function of macrophages, orderly secretion of cytokines, and protection of microenvironment balance in the injured tissues. HDC-driven macrophage reprogramming ensures the simultaneous release of various cytokine complementarity, thus achieving spatio-temporal coordination of various angiogenesis regulatory mechanisms. In conclusion, the results of this study demonstrate the diverse functions of macrophages and HDC in ischemic conditions, suggesting novel therapeutic targets and approaches for managing ischemic diseases.

Prior research has demonstrated that intraperitoneal or subcutaneous injection of exogenous histamine can alleviate myocardial injury caused by acute myocardial infarction or chemotherapy drugs in Hdc^-/-^ mice 54. Since HDC-expressing macrophages and neutrophils appear and produce histamine in the early stages of ischemic injury, the supplementation of appropriate exogenous histamine in the appropriate time window might be a new strategy for the intervention in ischemic injury. However, systemic application of histamine may cause side effects such as diarrhea, headache, nasal congestion, hypotension, arrhythmia, skin urticaria, and pruritus 55. Due to the characteristics of easy degradation, poor fat solubility, and difficult to function stably, how to stably encapsulate and accurately deliver histamine into cells is an urgent problem to be solved 56. Therefore, we intend to design a histamine sustained-release hydrogel to deliver histamine locally in ischemic muscle. Dopamine (DA)-cross linked hyaluronan (HA) hydrogel has exhibited the characteristics of good histocompatibility and long half-life could increase the retention and stability of histamine *in vivo* 29. The results of this study first demonstrate that HA-DA@histamine Hydrogel could significantly improve vascularization, reduce inflammatory responses, and recover limb function after HLI.

## Conclusion

In summary, this study offers ample evidence to support the significant contribution of bone marrow-derived HDC^+^ macrophages in the processes of angiogenesis and muscle regeneration following hindlimb ischemia. Histamine could promote endothelial cell migration and tubular formation mainly through the activation of H_1_R/CXCL/PI3K/AKT pathway. Knockout of HDC in macrophages inhibits endogenous histamine production and pro-angiogenesis factors expression, inducing a harsh microenvironment that is not conducive to the interaction between macrophages and endothelial cells. Thus, the HA-DA@histamine Hydrogel has been developed and shown to have potential as a noninvasive therapeutic option for treating ischemic muscle tissue injury by regulating inflammation and angiogenesis. In addition, the findings of this study indicate that the use of histamine receptor antagonists may affect the prognosis of peripheral artery diseases, which needs attention to optimize future clinical interventions.

## Supplementary Material

Supplementary figures and tables.
