# Supplementary material for: HDC/histamine Signaling Axis Drives Macrophage Reprogramming to Promote Angiogenesis in Hindlimb-Ischemic Mice
Source: Int J Biol Sci. 2025 Mar 19;21(6):2508–30. doi: 10.7150/ijbs.105148 (PMC12035888; doi:10.7150/ijbs.105148)
Supplement: Supplementary file 1 — Supplementary figures and tables. [file ijbsv21p2508s1.pdf]

# Supplementary Figures

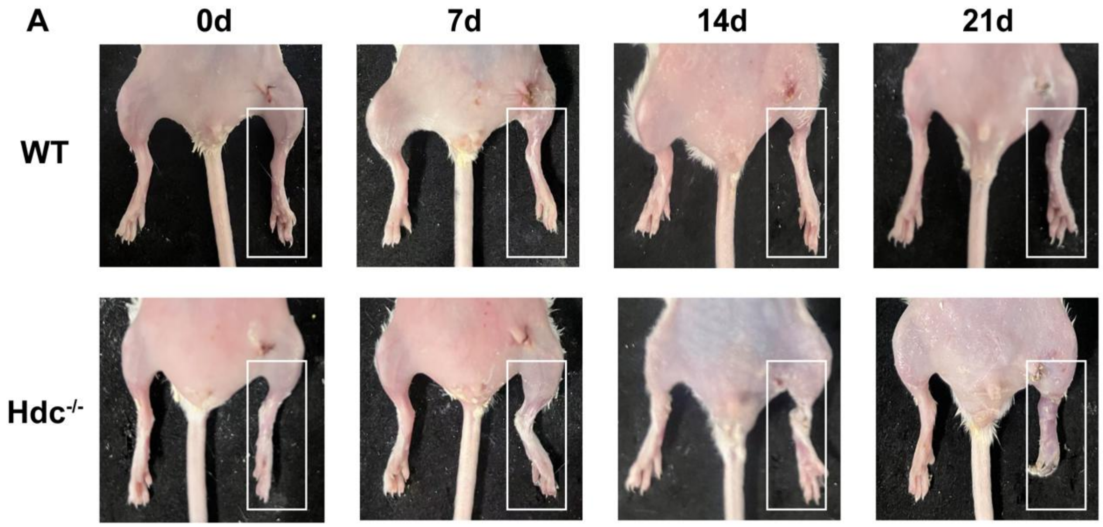


**Fig. S1. The Deletion of Hdc Impairs Hindlimb Ischemia (HLI)-Induced Revascularization.**

**(A)** A picture of the ischemic limb is shown at each point per group. Each photograph was taken under the same conditions (n=6).

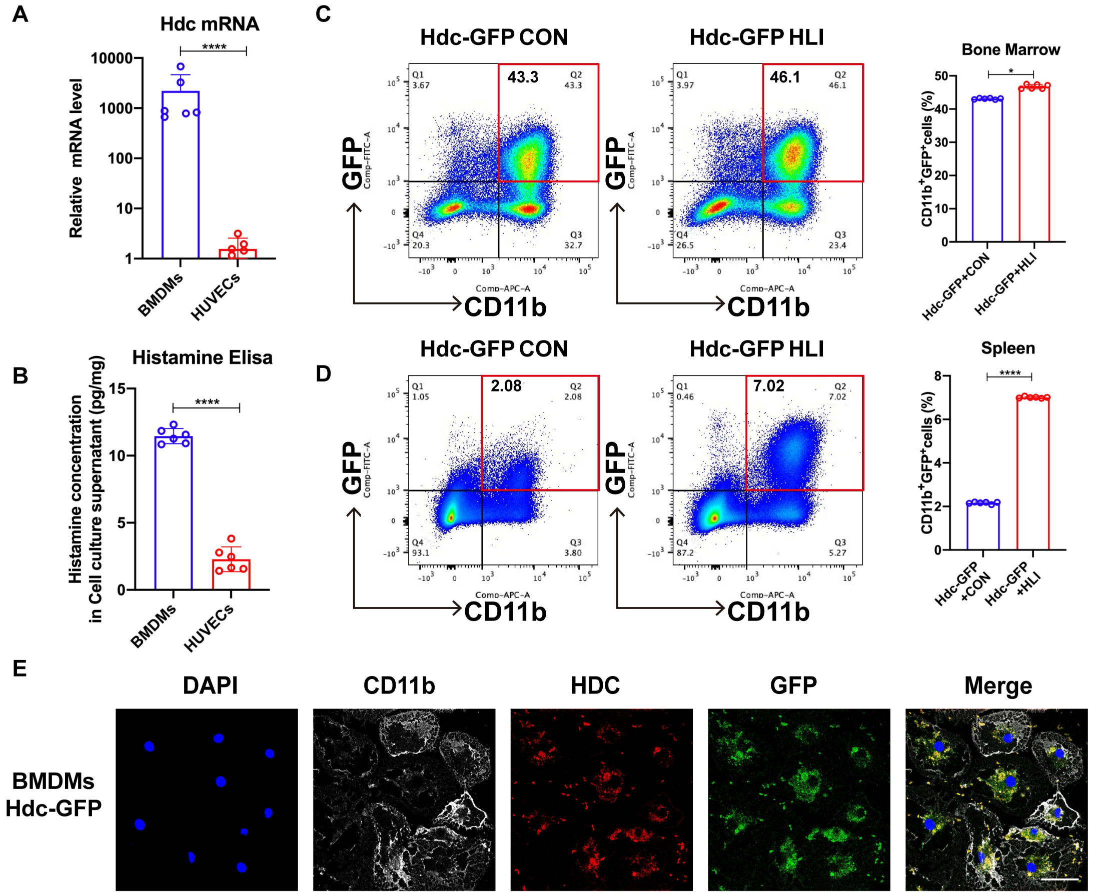


**Fig. S2. Bone marrow-derived macrophages (BMDMs) are the predominant HDC-expressing sites during HLI. (A)** RT-qPCR showing mRNA levels of HDC mRNA in BMDMs and HUVECs (n=6). **(B)** ELISA of secreted Histamine from BMDMs and HUVECs (n=6). **(C)** Representative images and quantification of FACS analysis of the $GFP^+$ cell percentage in the bone marrow of Hdc-$GFP^+$ mice before and 3 days after HLI (n=6). **(D)** Representative images and quantification of FACS analysis of the $GFP^+$ cell percentage in the spleen of Hdc-$GFP^+$ mice before and 3 days after HLI (n=6). **(E)** Representative images of HDC (red), GFP (green), CD11b (white) and DAPI (blue) immunostainings of bone marrow derived macrophages of Hdc-$GFP^+$ mice; Scale bar, 20μm. For all experiments, error bars represent the mean ± SD. *$P < 0.05$, **$P < 0.01$, ***$P < 0.001$, ****$P < 0.0001$

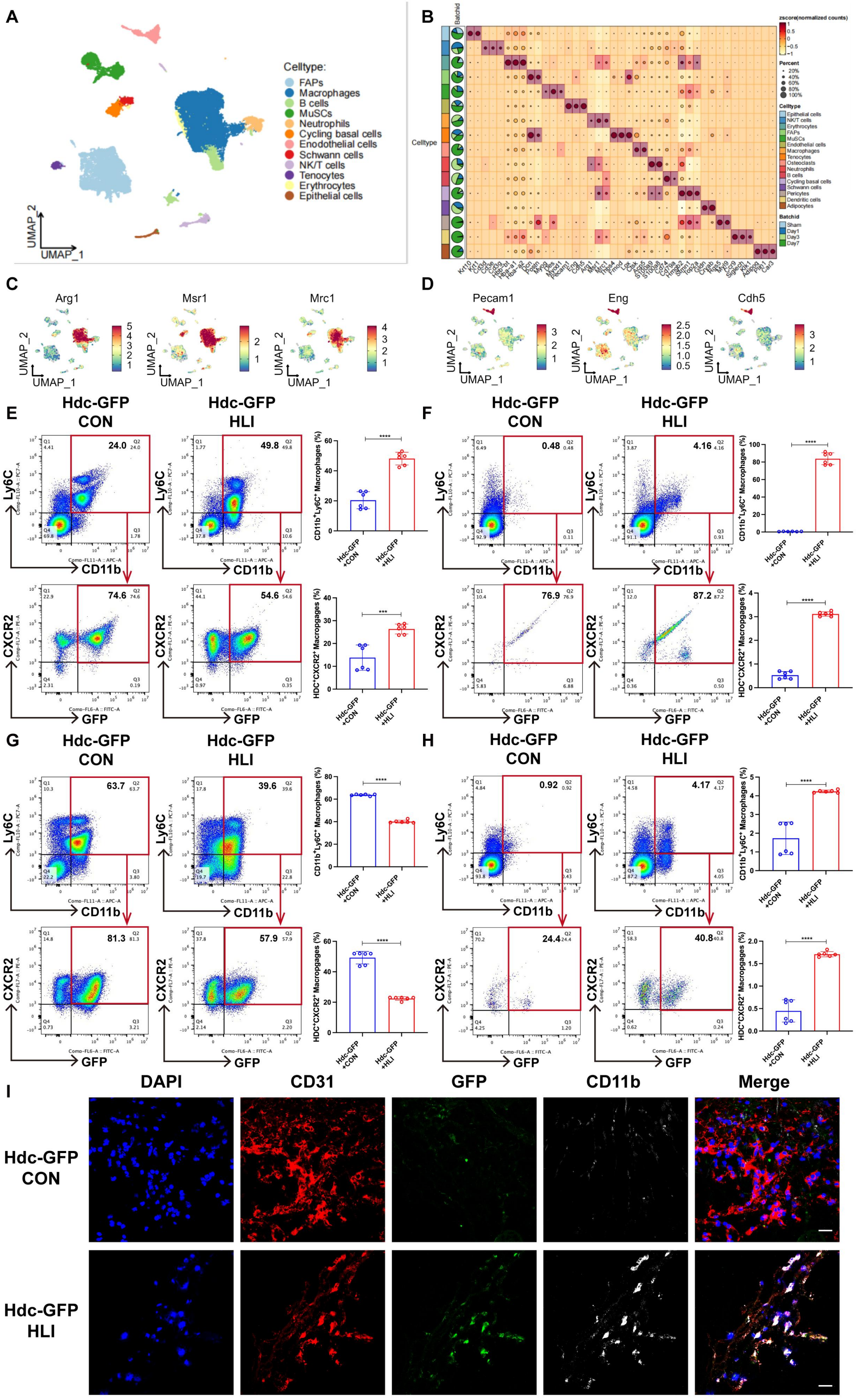
A
B
Celltype:
FAPs
Macrophages
B cells
MuSCs
Neutrophils
Cycling basal cells
Enodothelial cells
Schwann cells
NK/T cells
Tenocytes
Erythrocytes
Epithelial cells
UMAP_2
UMAP_1
Batchid
Celltype
zscore(normalized counts)
Percent
Batchid
Sham
Day1
Day3
Day7
C
Arg1
Msr1
Mrc1
D
Pecam1
Eng
Cdh5
E
F
G
H
Hdc-GFP
CON
Hdc-GFP
HLI
Ly6C
CD11b
CXCR2
GFP
CD11b+Ly6C+ Macrophages (%)
HDC+CXCR2+ Macrophages (%)
Hdc-GFP +CON
Hdc-GFP +HLI
I
DAPI
CD31
GFP
CD11b
Merge
Hdc-GFP
CON
Hdc-GFP
HLI

**Fig. S3. Single-cell transcriptomics analysis suggests an association between $HDC^+$ macrophages and angiogenesis during the process of HLI (A)** Reference-based integration of skeletal muscle mononucleated cell datasets prepared from WT mice (GSE227075). **(B)** Dot plot of selected marker genes for each cluster and lineage in aggregate cell clusters. **(C)** Feature plot showing Arg1, Msr1, and Mrc1 gene expression in macrophages. **(D)** Feature plot showing Pecam1, Eng, and Cdh5 gene expression in endothelial cells. **(E)** Representative images and quantification of FACS analysis of the $HDC^+CXCR2^+$ cell percentage in the peripheral blood of Hdc-GFP mice before and 1 days after HLI (n=6). **(F)** Representative images and quantification of FACS analysis of the $HDC^+CXCR2^+$ cell percentage in the muscle tissue of Hdc-GFP mice before and 1 days after HLI (n=6). **(G)** Representative images and quantification of FACS analysis of the $HDC^+CXCR2^+$cell percentage in the bone marrow of Hdc-GFP$^+$ mice before and 1 days after HLI (n=6). **(H)** Representative images and quantification of FACS analysis of the $HDC^+CXCR2^+$cell percentage in the spleen of Hdc-GFP$^+$ mice before and 1 days after HLI (n=6). **(I)**Representative images of CD31 (red), Hdc-GFP (green), CXCR2 (White) and DAPI (blue) immunostainings of gastrocnemius muscle of Hdc-GFP mice before and 3 days after HLI; Scale bar, 50μm. For all experiments, error bars represent the mean ± SD. *P < 0.05, **P < 0.01, ***P < 0.001, ****P < 0.0001

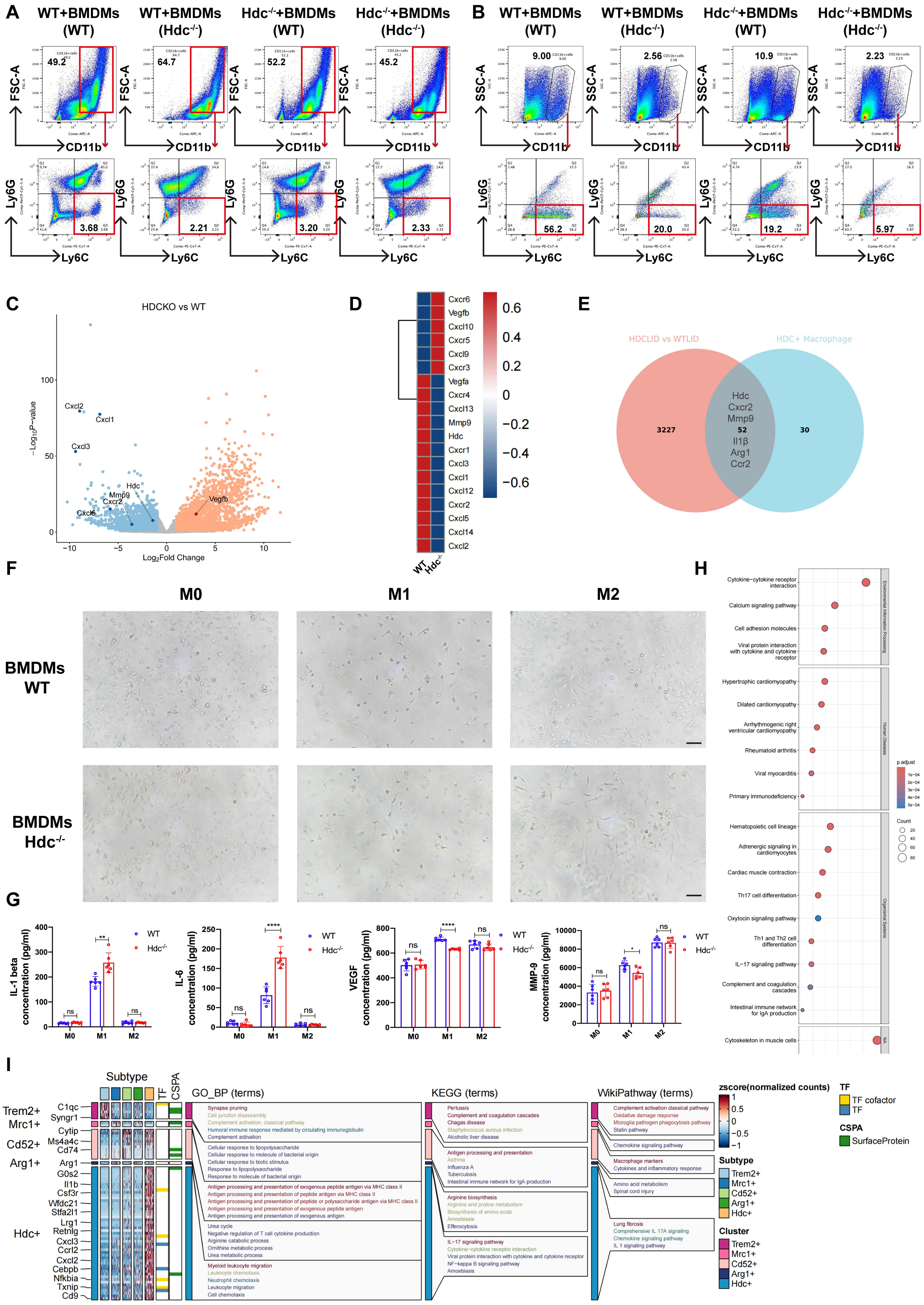
A
WT+BMDMs (WT)
WT+BMDMs (Hdc-/-)
Hdc-/-+BMDMs (WT)
Hdc-/-+BMDMs (Hdc-/-)
FSC-A
CD11b
Ly6G
Ly6C
49.2
64.7
52.2
45.2
3.68
2.21
3.20
2.33
B
SSC-A
9.00
2.56
10.9
2.23
56.2
20.0
19.2
5.97
C
HDCKO vs WT
−Log10P−value
Log2Fold Change
Cxcl2
Cxcl1
Cxcl3
Hdc
Mmp9
Cxcr2
Cxcl6
Vegfb
D
Cxcr6
Vegfb
Cxcl10
Cxcr5
Cxcl9
Cxcr3
Vegfa
Cxcr4
Cxcl13
Mmp9
Hdc
Cxcr1
Cxcl3
Cxcl1
Cxcl12
Cxcr2
Cxcl5
Cxcl14
Cxcl2
WT
Hdc-/-
E
HDCLID vs WTLID
HDC+ Macrophage
3227
Hdc
Cxcr2
Mmp9
52
Il1β
Arg1
Ccr2
30
F
M0
M1
M2
BMDMs WT
BMDMs Hdc-/-
G
IL-1 beta concentration (pg/ml)
IL-6 concentration (pg/ml)
VEGF concentration (pg/ml)
MMP-9 concentration (pg/ml)
WT
Hdc-/-
ns
**
****
*
H
Cytokine−cytokine receptor interaction
Calcium signaling pathway
Cell adhesion molecules
Viral protein interaction with cytokine and cytokine receptor
Hypertrophic cardiomyopathy
Dilated cardiomyopathy
Arrhythmogenic right ventricular cardiomyopathy
Rheumatoid arthritis
Viral myocarditis
Primary immunodeficiency
Hematopoietic cell lineage
Adrenergic signaling in cardiomyocytes
Cardiac muscle contraction
Th17 cell differentiation
Oxytocin signaling pathway
Th1 and Th2 cell differentiation
IL−17 signaling pathway
Complement and coagulation cascades
Intestinal immune network for IgA production
Cytoskeleton in muscle cells
p.adjust
Count
I
Subtype
TF
CSPA
Trem2+
Mrc1+
Cd52+
Arg1+
Hdc+
C1qc
Syngr1
Cytip
Ms4a4c
Cd74
Arg1
G0s2
Il1b
Csf3r
Wfdc21
Stfa2l1
Lrg1
Retnlg
Cxcl3
Ccrl2
Cxcl2
Cebpb
Nfkbia
Txnip
Cd9
GO_BP (terms)
Synapse pruning
Cell junction disassembly
Complement activation, classical pathway
Humoral immune response mediated by circulating immunoglobulin
Complement activation
Cellular response to lipopolysaccharide
Cellular response to molecule of bacterial origin
Cellular response to biotic stimulus
Response to lipopolysaccharide
Response to molecule of bacterial origin
Antigen processing and presentation of exogenous peptide antigen via MHC class II
Antigen processing and presentation of peptide antigen via MHC class II
Antigen processing and presentation of peptide or polysaccharide antigen via MHC class II
Antigen processing and presentation of exogenous peptide antigen
Antigen processing and presentation of exogenous antigen
Urea cycle
Negative regulation of T cell cytokine production
Arginine catabolic process
Ornithine metabolic process
Urea metabolic process
Myeloid leukocyte migration
Leukocyte chemotaxis
Neutrophil chemotaxis
Leukocyte migration
Cell chemotaxis
KEGG (terms)
Pertussis
Complement and coagulation cascades
Chagas disease
Staphylococcus aureus infection
Alcoholic liver disease
Antigen processing and presentation
Asthma
Influenza A
Tuberculosis
Intestinal immune network for IgA production
Arginine biosynthesis
Arginine and proline metabolism
Biosynthesis of amino acids
Amoebiasis
Efferocytosis
IL−17 signaling pathway
Cytokine−cytokine receptor interaction
Viral protein interaction with cytokine and cytokine receptor
NF−kappa B signaling pathway
Amoebiasis
WikiPathway (terms)
Complement activation classical pathway
Oxidative damage response
Microglia pathogen phagocytosis pathway
Statin pathway
Chemokine signaling pathway
Macrophage markers
Cytokines and inflammatory response
Amino acid metabolism
Spinal cord injury
Lung fibrosis
Comprehensive IL 17A signaling
Chemokine signaling pathway
IL 1 signaling pathway
zscore(normalized counts)
TF
TF cofactor
TF
CSPA
SurfaceProtein
Subtype
Cluster

1

**Fig. S4. Hdc knockout induced atypical macrophage polarization and down-regulated pro-angiogenic factors expression during HLI by regulating NF-κB and MAPK pathways** **(A)** Representative images of FACS analysis of the macrophage's percentage in the peripheral blood of WT and $Hdc^{-/-}$ mice transplanted with bone marrow of each other 3 days after HLI. **(B)** Representative images of FACS analysis of the macrophage's percentage in the muscle tissue of WT and $Hdc^{-/}$- mice transplanted with bone marrow of each other 3 days after HLI. **(C)** Volcano plot of the expression difference between ischemia muscle tissue of WT mice and $Hdc^{-/-}$ mice 3 days after HLI. Red dots indicate transcripts that were increased (padj< 0.05, Log2 fold change > 1), whereas blue dots indicate decreased transcripts (padj< 0.05, Log2 fold change < 1). **(D)** Heat map of the expression difference between ischemia muscle tissue of WT mice and $Hdc^{-/-}$ mice 3 days after HLI. **(E)** Venn diagram showing the common expression difference between sc-RNA-seq before and after HLI in WT mice and RNA-seq in WT mice and $Hdc^{-/-}$ mice 3 days after HLI. **(F)** Optical microscope image of M0 Macrophages, M1 Macrophages, and M2 Macrophages collected from bone marrow of WT and $Hdc^{-/-}$ mice; Scale bar, 50μm. **(G)** Elisa of secreted IL-1β, Il-6, VEGFA, and MMP-9 from BMDMs of WT and $Hdc^{-/-}$ mice. BMDMs were treated with LPS/IFN-γ or IL-4/IL-13 for 24 h (n=6). **(H)** Heat map showing KEGG enrichment analyses of ischemia muscle tissue of WT mice and $Hdc^{-/-}$ mice 3 days after HLI. **(I)** Heat map showing GO enrichment analyses, KEGG enrichment analyses and Wiki Pathway of five macrophage subtypes. For all experiments, error bars represent the mean ± SD.*P < 0.05, **P < 0.01, ***P < 0.001, ****P < 0.0001.

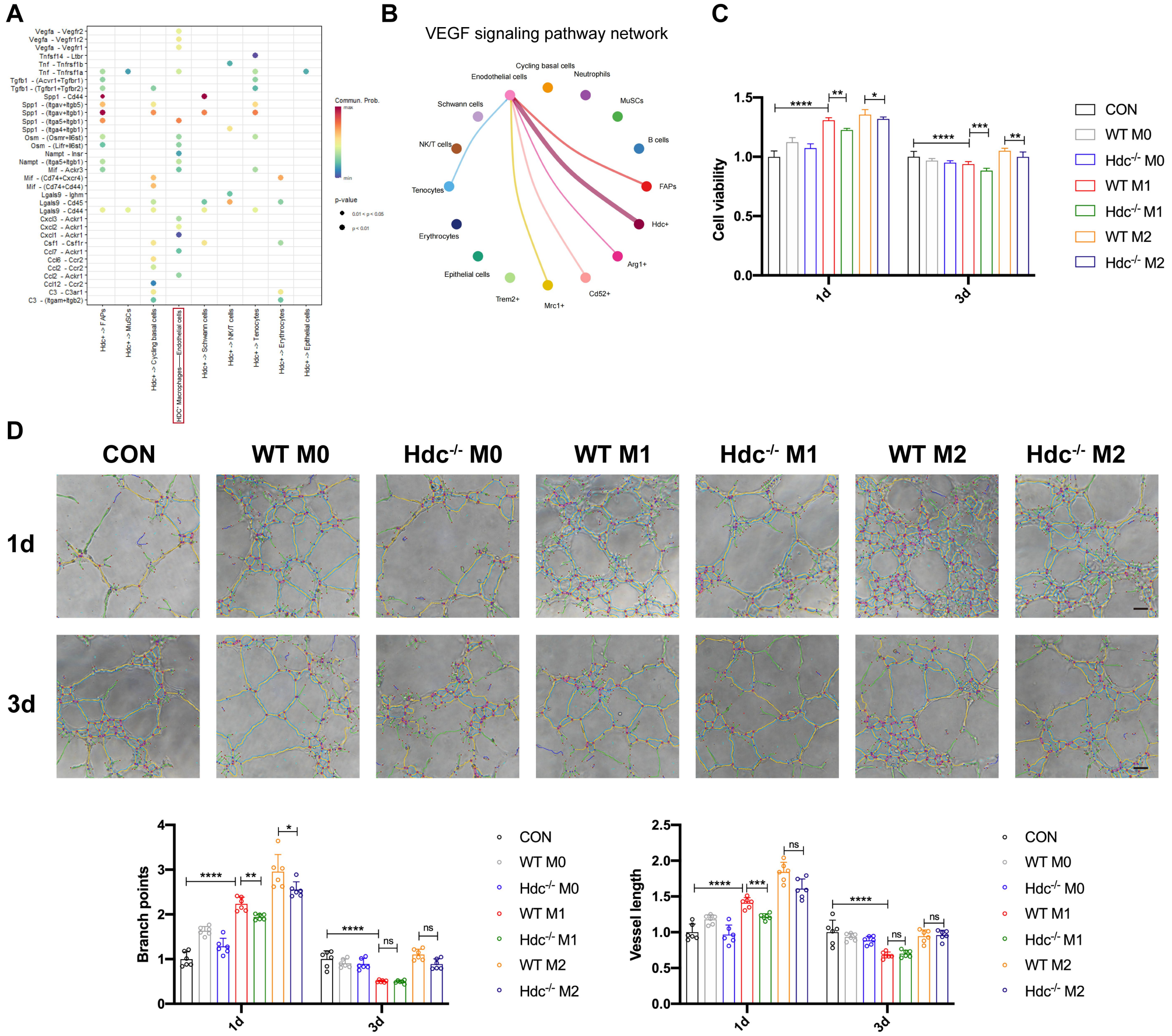


**Fig. S5. HDC$^+$ macrophages combine to endothelial cells via CXCR2-CXCL2 loop and promote angiogenesis mediated by VEGF, MMP-9, and IL-1β (A)** Heatmap of HDC$^+$ macrophage interacts with various types cells in skeletal muscle. **(B)** Cell chat showing HDC$^+$ macrophage interacts with various types cells though VEGF in skeletal muscle. Edges are scaled by the inferred regulatory potential of the interaction. **(C)** CCK8 cell proliferation test of HUVECs co-cultured with WT BMDM and Hdc-/- BMDM for 1 day and 3 days. HUVECs were pretreated with 10μM $H_2O_2$ and 1μm AST; BMDMs were treated with LPS/IFN-γ or IL-4/IL-13

for 24 h; Scale bars, 50μm; (n=6). **(D)** Representative images and quantification of tube formation assay of HUVECs co-cultured with WT BMDM and $Hdc^{-/-}$ BMDM for 1day and 3days. HUVECs were pretreated with 10μM $H_2O_2$ and 1μm AST; BMDMs were treated with LPS/IFN-γ or IL-4/IL-13 for 24 h; Scale bars, 50μm; (n=6). For all experiments, error bars represent the mean ± SD.*P < 0.05, **P < 0.01, ***P < 0.001, ****P < 0.0001.

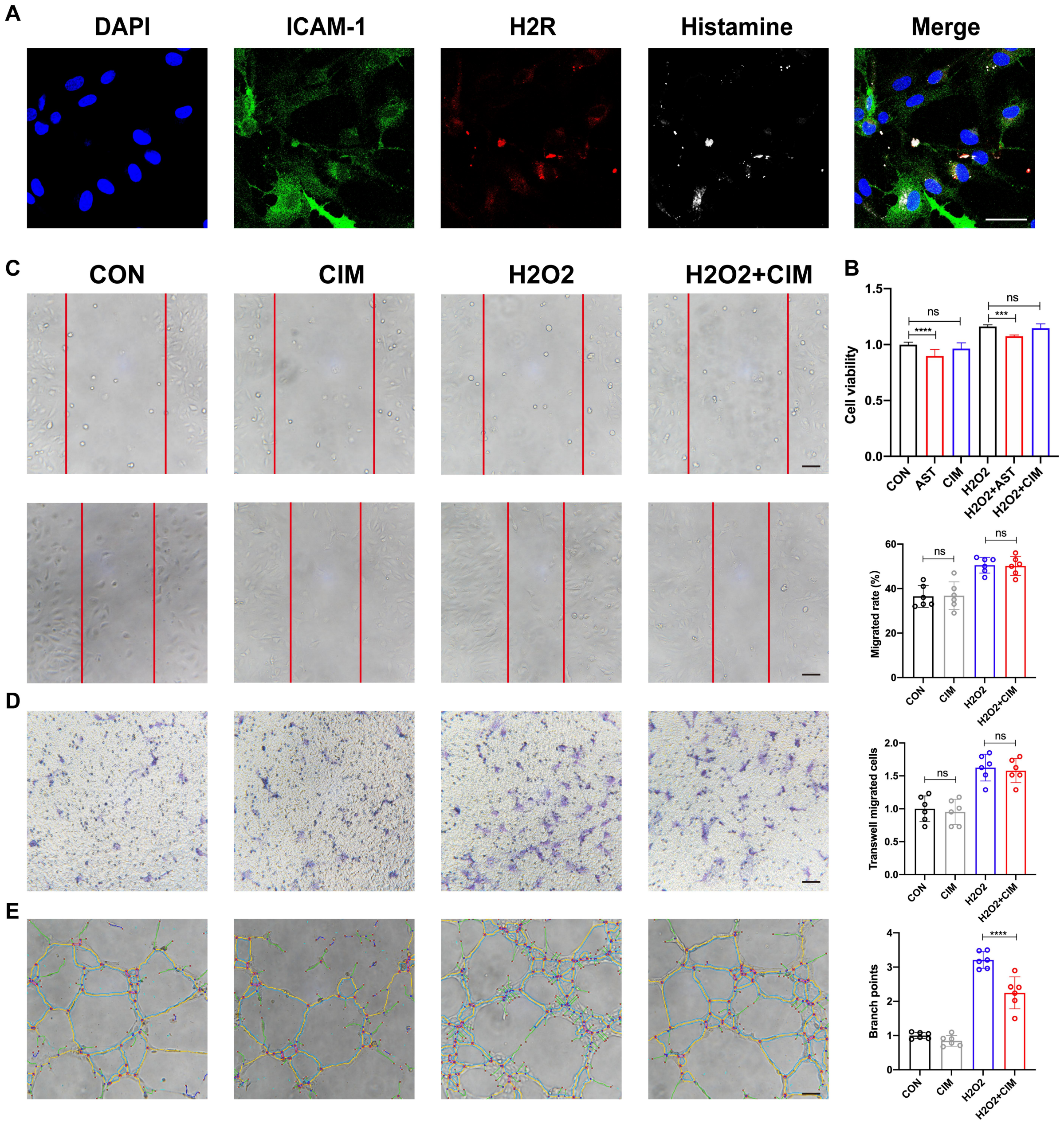


**Fig. S6. Histamine promotes endothelial cell migration and tube formation by activating $H_1R$ and CXCL/PI3K/AKT signaling pathway (A)** Representative images of ICAM-1(green), $H_2R$ (red), histamine (white) and DAPI (blue) immunostainings of HUVECs; scale bar, 20μm. **(B)** CCK8 cell proliferation test of HUVECs cultured in conditioned medium with or without 1μM AST or CIM. HUVECs were pretreated with 10μM $H_2O_2$. (n=6). **(C)** Representative

images and quantification of scratch wound healing assay of HUVECs cultured in conditioned medium with or without 1μM CIM. HUVECs were pretreated with 10μM $H_2O_2$. Scale bars, 50μm, (n=6). **(D)** Representative images and quantification of transwell assays of HUVECs cultured in conditioned medium with or without 1μM CIM. HUVECs were pretreated with 10μM $H_2O_2$. Scale bars, 50μm, (n=6). **(E)** Representative images and quantification of tube formation assay of HUVECs cultured in conditioned medium with or without 1μM CIM. HUVECs were pretreated with 10μM $H_2O_2$. Scale bars, 50 μm, (n=6). For all experiments,error bars represent the mean ± SD.*$P < 0.05$, **$P < 0.01$, ***$P < 0.001$, ****$P < 0.000$

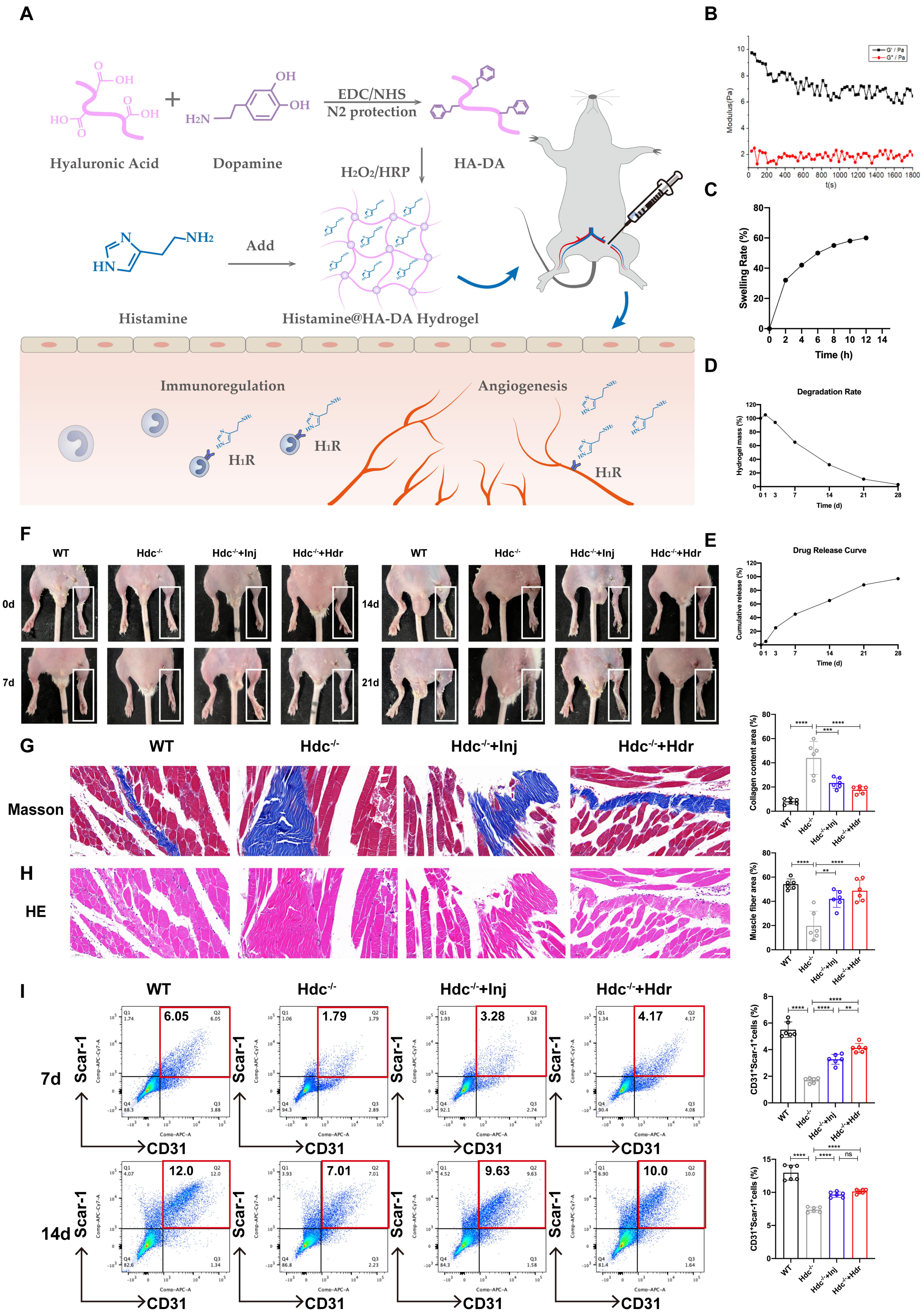
A
Hyaluronic Acid
Dopamine
EDC/NHS
N2 protection
HA-DA
H2O2/HRP
Histamine
Add
Histamine@HA-DA Hydrogel
Immunoregulation
Angiogenesis
H1R
B
G' / Pa
G" / Pa
Modulus(Pa)
t(s)
C
Swelling Rate (%)
Time (h)
D
Degradation Rate
Hydrogel mass (%)
Time (d)
E
Drug Release Curve
Cumulative release (%)
Time (d)
F
WT
Hdc-/-
Hdc-/-+Inj
Hdc-/-+Hdr
0d
7d
14d
21d
G
Masson
Collagen content area (%)
H
HE
Muscle fiber area (%)
I
7d
14d
Scar-1
CD31
6.05
1.79
3.28
4.17
12.0
7.01
9.63
10.0
CD31+Scar-1+cells (%)
ns

**Fig. S7. Histamine-delivering Hydrogel promotes skeletal muscle regeneration in Hdc$^{-/-}$ mice after HLI by regulating angiogenesis and inflammatory disorders (A)** Schematic illustration on gene therapy strategy of HA-DA@histamine hydrogel or delivering histamine to ischemic limb to promote angiogenesis, reduce ischemia-induced muscle damage and restore limb function. **(B)** Rheological properties of HA-DA@histamine hydrogel at 37°C. **(C)** Swelling rate of HA-DA@histamine hydrogel at 37°C in PBS. **(D)** Degradation rate of HA-DA@histamine hydrogel at 37°C in PBS. **(E)** The release rate of histamine from HA-DA@histamine hydrogel at 37°C in PBS. **(F)** A picture of the ischemic limb is shown at each point per group. Each photograph was taken under the same conditions. **(G)** Representative images and quantitative analysis of Masson' s staining of injured gastrocnemius muscle from each group at day 21 post injury in ischemic muscles (n=6); Scale bar, 50μm. **(H)** Representative images and quantitative analysis of H&E staining of injured gastrocnemius muscle from each group at day 21 post-injury in ischemic muscles (n=6); Scale bar, 50μm. **(I)** Representative flow cytometry plots with quantification of CD31$^+$ Sca-1$^+$ endothelial cells (n=6). For all experiments, error bars represent the mean ± SD.*P < 0.05, **P < 0.01, ***P < 0.001, ****P < 0.0001.

# Supplementary Tables

## Table S1. Animals (in vivo studies)

| Species | Vendor or Source | Background Strain |
|---|---|---|
| Wide type mice | Department of Laboratory Animal Science at Fudan University | Balb/C |
| Hdc$^{-/-}$ mice | Supplied by Professor Timothy C. Wang | Balb/C |
| Hdc-GFP mice | Supplied by Professor Timothy C. Wang | Balb/C |

**Table S2. Fluorochrome-conjugated antibodies used in flow cytometry analysis**

| Experiments | Antibodies | Cat No. | Company |
|---|---|---|---|
| Macrophage identification and polarization | CD45 | 147709 | Biolegend |
| | CD11b | 101211 | Biolegend |
| | Ly6C | 128018 | Biolegend |
| | Ly6G | 127616 | Biolegend |
| | F4/80 | 123110 | Biolegend |
| | CD86 | 374207 | Biolegend |
| | CD206 | 321121 | Biolegend |
| Endothelial cells and angiogenesis | CD31 | 102410 | Biolegend |
| | Scar-1 | 108125 | Biolegend |

**Table S3. Primary antibodies used in experiments**

| Experiments | Antibodies | Cat No. | Company |
|---|---|---|---|
| Immunofluorescence and immunohistochemistry assay | CD31 | ab182981 | Abcam |
| | CD68 | ab283654 | Abcam |
| | α-SMA | ab179467 | Abcam |
| Western blot | CXCR-2 | A3301 | Abcronal |
| | ERK | #4695 | Cell Signaling Technology |
| | p-ERK | #4370 | Cell Signaling Technology |
| | iKBα | #4812 | Cell Signaling Technology |
| | p-iKBα | #2859 | Cell Signaling Technology |
| | PI3K(p85) | ab191606 | Abcam |
| | AKT | #4691 | Cell Signaling Technology |
| | p-AKT | #4060 | Cell Signaling Technology |

**Table S4. Primary antibodies used in experiments**

| Gene | Sequence 5’-3’ | Species |
|---|---|---|
| Gapdh | F: CCACTCACGGCAAATTCAAC<br>R: GTAGACTCCACGACATACTCAG | |
| Hdc | F: TGCCTGTGTTTGTCTGTGCAACG<br>R: ATCTGCCAATGCATGAAGTCCGTG | |
| $H_1R$ | F: GCCTGGTTTCTCTCCTTCCT<br>R: TGAGCAAAGTGGGGAGGTAG | |
| Il1β | F: ACTCATTGTGGCTGTGGAGA<br>R: TTGTTCATCTCGGAGCCTGT | |
| Il6 | F: CTGGGGATGTCTGTAGCTCA<br>R: CTGTGAAGTCTCCTCTCCGG | |
| Arg-1 | F: CTGAGCTTTGATGTCGACGG<br>R: TCCTCTGCTGTCTTCCCAAG | *Mus musculus* |
| Mrc-1 | F: TGGATGGATGGGAGCAAAGT<br>R: GCTGCTGTTATGTCTCTGGC | |
| Vegfa | F: TCTCCTTCCTCTCTATTCACCT<br>R: CATCCACCAGTCCATATACCTC | |
| Vegfb | F: CTATGACCGATTCCTGTCAGTC<br>R: CGTGATGAGAAAGTACCAGTTG | |
| Mmp-9 | F: TGGGCGTTAGGGACAGAAAT<br>R: GAACCATAACGCACAGACCC | |
| Gapdh | F: GGCTGTTGTCATACTTCTCATGG<br>R: GGCTGTTGTCATACTTCTCATGG | |
| Hdc | F: ATGCACGCCTACTACCCAG<br>R: CAGTCCATGACGTTCATCTCC | |
| $H_1R$ | F: AGATGTGTGAGGGCAACAAGA<br>R: CAAGCAGATAGTGCTCAGGAC | |
| Vcam-1 | F: GGGAAGATGGTCGTGATCCTT<br>R: TCTGGGGTGGTCTCGATTTTA | |
| Cxcl-1 | F: ACTCTACCTGCACACTGTCC<br>R: TCCCCTGCCTTCACAATGAT | |
| Cxcl-2 | F: CGCCCAAACCGAAGTCATAG<br>R: CTCTGCAGCTGTGTCTCTCT | *Homo sapiens* |
| Cxcl-5 | F: AGCTGCGTTGCGTTTGTTTAC<br>R: TGGCGAACACTTGCAGATTAC | |
| Cxcl-9 | F: CCAGTAGTGAGAAAGGGTCGC<br>R: AGGGCTTGGGGCAAATTGTT | |
| Cxcl-10 | F: GTGGCATTCAAGGAGTACCTC<br>R: TGATGGCCTTCGATTCTGGATT | |
| Cxcl-12 | F: ATTCTCAACACTCCAAACTGTGC<br>R: ACTTTAGCTTCGGGTCAATGC | |
| Cxcl-13 | F: GCTTGAGGTGTAGATGTGTCC<br>R: CCCACGGGGCAAGATTTGAA | |

**Table S5. Result of Mendelian randomization (MR)**

| Outcome | Exposure | Method | nsnp | b | se | pval |
|---|---|---|---|---|---|---|
| Sequel of lower limb injuries | Ant-ihistamine medication | MR Egger | 10 | 0.653251919 | 0.455050691 | 0.189049289 |
| Sequel of lower limb injuries | Anti-histamine medication | Weighted median | 10 | 0.466976953 | 0.195508223 | 0.016915998 |
| Sequel of lower limb injuries | Anti-histamine medication | Inverse variance weighted | 10 | 0.353664617 | 0.152956643 | 0.020767288 |
| Sequel of lower limb injuries | Anti-histamine medication | Simple mode | 10 | 0.4745811 | 0.3127703 | 0.163489992 |
| Sequel of lower limb injuries | Anti-histamine medication | Weighted mode | 10 | 0.500100985 | 0.310773482 | 0.142030465 |

## Table S6. Horizontal pleiotropy tests of Mendelian randomization (MR)

| Outcome | Exposure | egger_intercept | se | pval |
|---|---|---|---|---|
| Sequel of lower limb injuries | Anti-histamine medication | -0.025768518 | 0.036863073 | 0.504343372 |

## Table S7. Pleiotropy test of Mendelian randomization (MR)

| Outcome | Exposure | Method | Q | Q_df | Q_pval |
|---|---|---|---|---|---|
| Sequel of lower limb injuries | Anti-histamine medication | MR Egger | 4.558602914 | 8 | 0.803541838 |
| Sequel of lower limb injuries | Anit-ihistamine medication | Inverse variance weighted | 5.047250567 | 9 | 0.830170001 |
